# Foliar Endophytic Fungal Communities Are Driven by Leaf Traits—Evidence From a Temperate Tree Diversity Experiment

**DOI:** 10.1002/ece3.71691

**Published:** 2025-07-17

**Authors:** Michael Köhler, Pablo Castro Sánchez‐Bermejo, Georg Hähn, Olga Ferlian, Nico Eisenhauer, Tesfaye Wubet, Sylvia Haider, Helge Bruelheide

**Affiliations:** ^1^ Institute of Biology/Geobotany and Botanical Garden Martin Luther University Halle‐Wittenberg Halle (Saale) Germany; ^2^ German Centre for Integrative Biodiversity Research (iDiv) Halle‐Jena‐Leipzig Leipzig Germany; ^3^ Institute of Ecology Leuphana University of Lüneburg Lüneburg Germany; ^4^ BIOME Lab, Department of Biological, Geological and Environmental Sciences Alma Mater Studiorum University of Bologna Bologna Italy; ^5^ Institute of Biology Leipzig University Leipzig Germany; ^6^ Department of Community Ecology Helmholtz Centre for Environmental Research –UFZ Halle Germany

**Keywords:** biodiversity‐ecosystem functioning experiment, fungal endophytes, leaf spectrometry, leaf traits, metagenomics, within‐individual trait variation

## Abstract

Fungal endophyte communities are mainly driven by host plant identity and geographic location. However, little is known about interactions between endophytes and characteristics of the host plant such as leaf functional traits, which vary both among and within host species. Previous studies focused on a limited number of host plant species and did not control for varying conditions in the host's neighborhood, which affect leaf functional traits and, in turn, might affect fungal endophyte communities. Using a tree diversity experiment in which all trees grow under standardized conditions, we were able to assess the contributions of host tree identity, host neighborhood species richness, and host community composition as well as the variation of leaf traits caused by these factors on taxonomic richness and community composition of foliar fungal endophytes. We used next‐generation amplicon sequencing to analyze the fungal endophyte community and visible–near infrared spectrometry data to predict the mean values and the intra‐individual variation of leaf traits in individual trees. We found both mean trait values and intra‐individual trait variation to have significant effects on endophyte richness. Mean trait values of leaf dry matter content, leaf carbon, leaf nitrogen, and leaf carbon‐to‐nitrogen ratio exhibited negative effects on endophyte richness, whereas specific leaf area and leaf phosphorus content increased endophyte richness. Additionally, intra‐individual leaf‐trait variation generally had positive effects on richness. Overall endophyte community composition was influenced by mean leaf dry matter content and specific leaf area. Ascomycota were influenced by the specific leaf area, whereas Basidiomycota responded to leaf dry matter content. We demonstrate that functional leaf traits affect foliar endophyte communities, with positive diversity effects of host leaf nutrients that are essential, and likely limiting, for fungal endophytes. Although our study emphasizes the role of leaf traits in shaping fungal communities, we also acknowledge that these dynamic interactions could lead to traits being influenced by microbes through microbe–plant interactions.

## Graphical abstract

Our study demonstrates that functional leaf traits play a crucial role in shaping fungal endophyte richness and community composition, even beyond the effects of tree species identity. Traits associated with the leaf economics spectrum exhibited both positive and negative relationships with endophyte richness depending on specific trait and taxa combinations, whereas intra‐individual variation in leaf traits had a generally positive relationship with endophyte richness by creating heterogeneous microhabitats within the tree crown. Additionally, fungal community composition responded primarily to specific leaf area and leaf dry matter content, with Ascomycota and Basidiomycota exhibiting distinct trait associations, highlighting the complexity of endophyte–plant interactions.

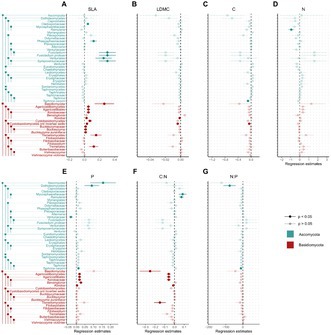

## 1 Introduction

Research on plant–microbe interactions has demonstrated an overall benefit of mutualistic host‐associated microbes for plant fitness and productivity (Vorholt 2012; Vandenkoornhuyse et al. 2015). It has been proposed that host‐associated microbes play a pivotal role by affecting their hosts fitness (Bringel and Couée 2015; Müller et al. 2016). For example, leaf‐associated microbes have been shown to alter secondary metabolite composition of host leaves (Li et al. 2023) and modulate photosynthesis or stomatal conductance (Sandy et al. 2023). Additionally, these microbes can enhance nutrient acquisition, including the uptake of foliar nitrogen (Christian et al. 2019). However, that plant–microbe interactions are bidirectional, as host plants have a strong influence on microbial community structure and composition (Vandenkoornhuyse et al. 2015). Using a tree biodiversity–ecosystem functioning (BEF) experiment, Laforest‐Lapointe et al. (2017) showed that leaf bacterial diversity was positively linked to productivity, even after accounting for direct effects of tree diversity. In BEF experiments, the number of host species is manipulated whilst standardizing confounding factors, such as varying tree density or abiotic conditions, which enables investigation of a generalized response to tree diversity (Bruelheide et al. 2014). BEF experiments have shown that plant‐microbe interactions are strongly driven by plant diversity (Latz et al. 2012; Tilman et al. 2012; Lange et al. 2015; Liang et al. 2016). However, results on testing the relationship between host species richness and fungal endophyte richness are equivocal. Endophyte richness was shown to be either unrelated to (Kambach et al. 2021) or negatively affected (Griffin et al. 2019) by local host tree species richness. In contrast, host species identity proved to be the most influential factor shaping endophyte communities (Liu et al. 2019; Kambach et al. 2021).

In the strictest sense, endophytic organisms are defined as those that do not induce any visual damage to their host (Petrini et al. 1991). Foliar endophytic fungi are, next to bacteria and protists, one of the main groups of foliar endophytic organisms (Petrini et al. 1991; Ploch et al. 2016; Yang et al. 2023). With very few exceptions, they have been detected in every plant analyzed so far, ranging from mosses and liverworts (Pocock and Duckett 1984; Zhang et al. 2013) to ferns (Younginger et al. 2023), gymnosperms, and angiosperms, including highly reduced parasitic plants (Ikeda et al. 2016; Kambach et al. 2021). Foliar endophytic fungi have been encountered in all types of habitats, from the extreme arctic to the humid tropics and drylands (Arnold et al. 2003; Zhang et al. 2013; Massimo et al. 2015). Colonizing such a diverse array of ecosystems, foliar fungal endophytes do not represent a phylogenetically distinct group, compared to, for example, arbuscular mycorrhiza, which are mainly formed by Glomeromycota (Parniske 2008). Although some classes of endophytic fungi seem to be the most prevalent groups of endophytes, for example, Dothideomycetes and Cystobasidiomycetes, it is unclear how endophytic communities as well as specific taxa respond to leaf traits and if these responses are mirrored in the endophytes' phylogenetic relatedness.

Many host plant species differ in their chemical and morphological leaf traits. However, the impact of leaf functional traits on endophytic fungi is, similarly to the effect of host species richness, only known for a very limited set of host species, and results are contradictory (Yang et al. 2016; Tellez et al. 2022). Several studies have described the leaf economics spectrum (LES) to be of potential importance for shaping fungal endophyte community composition (Kembel and Mueller 2014; Liu et al. 2019). The LES reflects a gradient in the resource‐use strategy, characterized by a trade‐off between a leaf's high photosynthetic rate, related to an acquisitive growth strategy, and a leaf's long lifespan, related to a conservative strategy (Wright et al. 2004; Díaz et al. 2016). Acquisitiveness is commonly associated with high specific leaf area (SLA) and high nutrient concentrations, in particular of nitrogen and phosphorus, which in turn are commonly associated with less investment in leaf construction and durability (Osnas et al. 2013).

The LES might serve as a predictive framework across plant species and environmental contexts by characterizing the trait combination describing the endophytic habitat (Kembel and Mueller 2014; Liu et al. 2019). Although there is no evidence yet that the LES gradient is associated with different types of endophyte communities, some studies revealed a strong relationship between the different types of photosynthesis mechanisms. For example, Tellez et al. (2022) described that endophyte communities in bromeliads with a Crassulacean acid metabolism differed significantly from those occurring in C_3_ bromeliads. In addition, there was a clear relationship to leaf sclerophylly. However, almost nothing is known about the effects of individual traits on endophyte taxon diversity, and patterns seem to be idiosyncratic, being highly dependent on host plant species and environmental conditions. For example, it was shown that the leaf carbon‐to‐nitrogen ratio (C:N) was positively correlated with fungal endophyte richness in *Vaccinium ovatum* but not in *Pinus muricata* (Oono et al. 2020). Further, endophyte diversity was shown to be negatively related to leaf mass per area (LMA, which is the inverse of SLA), leaf dry matter content (LDMC), leaf toughness, leaf thickness, and leaf carbon content (Tellez et al. 2022). Others encountered positive effects of LMA, leaf toughness (Molinari and Knight 2010), and leaf carbon content (Yang et al. 2016). Similarly contrasting results were reported for the effect of leaf nitrogen content on endophyte richness, for which some studies found an overall positive effect (Tellez et al. 2022), whereas other studies described negative effects (Rasmussen et al. 2007; Molinari and Knight 2010; Meng et al. 2022).

Leaves of individual plants, in particular those of trees, are exposed to vertical gradients in light and water availability (Proß et al. 2024), which affect endophytes indirectly by varying leaf trait expressions and directly by altering the microclimatic conditions. For example, both leaf traits and endophytes are significantly influenced by shade (Davitt et al. 2010; Williams et al. 2020). However, disentangling how shade is influencing leaf traits and as such directly and indirectly fungal endophytes requires an experimental setting and measurements of the micro‐environment within plants.

The aforementioned variation of abiotic factors as well as biotic interactions such as competition foster variation of leaf traits within individual trees (Castro Sánchez‐Bermejo, Monjau, et al. 2024). Intra‐individual leaf trait variation decreases with tree diversity (Tobias Proß et al. 2024; Castro Sánchez‐Bermejo, Carmona, et al. 2024; Castro Sánchez‐Bermejo, Monjau, et al. 2024), which could potentially affect endophyte communities. It has been proposed that environmental heterogeneity promotes fungal endophyte diversity (Ben‐Hur and Kadmon 2020). As such, it can be assumed that the availability of more heterogeneous microhabitats across the tree canopy is one of the main drivers of endophyte community composition. Following this hypothesis, intra‐individual leaf trait variation can be assumed to promote endophyte taxon richness within an individual host plant. Similar positive effects of diverse microhabitats have already been reported from wood‐decaying and soil fungi (Park et al. 2021; Kolényová et al. 2024).

In addition to host species identity and leaf traits, community assembly mechanisms of fungal foliar endophytes might depend on further characteristics, which, however, are yet unknown. Such hidden characteristics might be reflected in the host plant's phylogeny (Tedersoo et al. 2013). As phylogenetic relationships have been shown to predict community assembly of vascular plant species (Cadotte et al. 2013; Chang and HilleRisLambers 2019), they might also help predicting the assembly of associated fungal communities (Tedersoo et al. 2013). Given the frequent host‐specificity observed in foliar fungi, one might expect some degree of evolutionary association between fungal taxa and their host plants (Hantsch et al. 2013; Rutten et al. 2021). Thus, leaf traits might not only affect leaf fungi at the taxon level, but also at higher taxonomic levels, such as families, orders or phyla. Furthermore, leaf traits, which characterize the endophytes' potential habitats, are not independent of plant phylogeny and thus show strong phylogenetic signals (Meireles et al. 2020; Ávila‐Lovera et al. 2023). Understanding the taxonomic level at which relationships between leaf traits and fungal foliar endophytes emerge can provide valuable insights into the evolutionary history of plant–endophyte interactions. Since leaf traits exhibit strong phylogenetic signals across the plant kingdom and play a pivotal role in defining the habitat of fungal endophytes, these phylogenetic signals are likely reflected within the fungal endophyte community. Importantly, such patterns are most likely to emerge at lower fungal taxonomic levels, as closely related fungal taxa often share similar ecological niches and functional adaptations, which may be shaped by their host plant's traits (U'Ren et al. 2012; Nguyen, Song, et al. 2016). This is consistent with the patterns of host–symbiont co‐diversification observed in other fungal systems (Kembel and Mueller 2014; Feijen et al. 2018) and underscores the evolutionary coupling between plant traits and their associated fungal communities.

To explore the effects of LES traits and their intra‐individual variation on endophyte richness and community composition across gradients of tree diversity, we employed a novel approach combining next‐generation sequencing with visible–near infrared spectrometry on the same leaf samples. This unique dataset encompassed 227 trees representing eight native deciduous tree species within the MyDiv tree diversity experiment in central Germany (Ferlian et al. 2018).

On the level of individual trees, we tested the hypothesis (H1) that leaf traits with high values on the conservative side of the LES, such as high LDMC and leaf carbon content, are associated with lower endophyte taxon richness and a distinct community composition compared to those with high values on the acquisitive side, such as high SLA, leaf nitrogen content, and leaf phosphorus content. Furthermore, we hypothesized (H2) that, beyond the mean trait values of a tree, the intra‐individual trait variation in leaf traits within the same tree positively influences endophyte taxon richness. Finally, we expected (H3) that the fungal endophytes niches with respect to leaf traits have evolved more recently. This would be expected under the assumption of co‐evolution between host and endophytes and with a more recent divergence of leaf traits. Therefore, we hypothesized that niche differentiation is more pronounced at the tips of the phylogeny, that is, at lower taxonomic levels of fungal endophytes (such as family and genus) than at higher levels (such as phylum and order). Although our study is not able to test for co‐evolution, we assess a potential consequence of co‐evolution by examining whether niche differentiation varies across taxonomic levels.

## 2 Materials and Methods

### 2.1 Experimental Design

This study was conducted in the MyDiv experiment, a biodiversity–ecosystem functioning (BEF) experiment, located at the Bad Lauchstädt Experimental Research Station of the Helmholtz Centre for Environmental Research—UFZ in central Germany. This site is characterized by a temperate, continental climate with a mean annual precipitation of 484 mm and an annual mean temperature of 8.8°C (Ferlian et al. 2018). The experimental site had been used as agricultural land until 2012 and was afterward converted to grassland, before it was plowed to prepare for planting the experiment.

The experiment includes 80 plots established in March 2015. Each plot has a size of 11 m x 11 m and consists of 140 trees that were planted at a distance of 1 m. At the time of sampling, the trees were 8–9 years old. A species pool of 10 deciduous tree species was chosen for this experiment, with 5 of them predominantly associated with either arbuscular mycorrhizal (AM) or ectomycorrhizal (EM) fungi. Trees were planted in a tree species richness gradient of monocultures, two‐species, and four‐species mixtures with changing species compositions of the same host mycorrhizal types (AM or EM). Additionally, mixtures of AM and EM trees were established in the two‐ and four‐species mixtures. Thus, each plot is either of monotypic (AM or EM) or mixed (AM and EM) mycorrhizal status, resulting in a total of eight diversity levels as a combination of species richness and the mixture of mycorrhiza types (Ferlian et al. 2018).

We selected four tree species of each mycorrhizal type. *Acer pseudoplatanus* L., *Fraxinus excelsior* L., *Prunus avium* L. (L.), and *Sorbus aucuparia* L. were the chosen species associated with AM, whereas *Betula pendula* Roth, *Carpinus betulus* L., *Fagus sylvatica* L., and *Quercus petraea* (Matt.) Liebl. represented EM species. The preferential mycorrhizal types of host species were validated by sequencing and morphological root assessments (Heklau et al. 2021).

Sampling took place from August to September 2021. Samples were taken from a set of four adjacent trees, a tree species quartet (TSQ). The four individual trees belonged either to one, two, or four different tree species, respectively. We established two TSQs per plot, which preferably did not overlap and were located within the core area (8 × 8 m) of each plot. This resulted in 227 sampled trees from 39 plots in 30 species combinations (Appendix Table S1). Along a vertical gradient spanning the whole canopy within the crown interaction zone of the four TSQ partners, we collected five leaves from each tree (for details see Castro Sánchez‐Bermejo et al. 2024), which were then pooled into one sample per tree. All sampled leaves were immediately stored at 4°C–8°C in the field and further processed within 8 h.

### 2.2 Leaf Trait Measurements

In order to quantify the effect of leaf traits on endophytes, we used morphological and chemical traits measured by Castro Sánchez‐Bermejo, Monjau, et al. (2024) (Figure S1). Leaf reflectance across the radiation spectrum from 350 to 2500 nm was acquired for each leaf collected using visible–near infrared spectrometry (ASD “FieldSpec4” Wide‐Res Field Spectroradiometer; Malvern Panalytical Ltd., Almelo, the Netherlands). Then, by using convolutional neural networks (CNNs), five leaf traits from the LES were predicted from the spectral data: SLA, LDMC, leaf carbon content (C), leaf nitrogen content (N), and leaf phosphorus content (P) (Figure S1). In order to train the CNNs for leaf trait prediction, an independent set of 200 leaf samples was collected during the same fieldwork campaign (hereafter referred to as calibration set). Immediately after sampling, the fresh leaves of the calibration set were weighed and scanned with a resolution of 300 dpi. The leaf area from the scans was analyzed using the WinFOLIA software (Regent Instruments, Quebec, Canada). To determine dry weight, the leaves were dried for 72 h at 60°C and then reweighed. From this, both LDMC and SLA were calculated. The dried leaves were then ground into powder and P was determined using a spectrophotometric assay with the acid molybdate technique, whereas C, N, and the C:N ratio were analyzed using an elemental analyzer (Vario EL Cube, Elementar, Langenselbold, Germany). The predictive ability of the CNNs was assessed by using the coefficient of determination (*R* ^2^) for the predicted and measured trait values in a test set comprising 30% of the samples in the calibration set. *R* ^2^ was 0.75 on average, being highest for SLA (*R* ^2^ = 0.88) and lowest for C (*R* ^2^ = 0.65). Additionally, the ratios of N:P and C:P were calculated from the mean trait values of the respective traits (Figure S1).

### 2.3 DNA Extraction, Library Preparation, and Illumina Sequencing

DNA extraction, library preparation, and Illumina Sequencing followed Köhler et al. (2025). In short, leaves were subjected to leaf‐surface sterilization after leaf reflectance acquisition to remove all epiphyllous taxa, residuals, and contaminants following the protocol of Guerreiro et al. (2018). Afterward, we stamped five leaf discs with a 6 mm diameter per leaf, resulting in 25 leaf discs per sample with a total area of 706.86 mm^2^. DNA extraction was carried out using the Chargeswitch gDNA plant Kit (Invitrogen, Thermo Fisher Scientific, Dreieich, Germany) downscaled to 40% of the manufacturer's original reaction volume to account for the low sample weight, followed by amplification of the fungal Internal Transcribed Spacer 1 (ITS1) gene region located between 18S and the 5.8S rRNA gene (White 1990) using the primer pair ITS1‐F and ITS2R (Zhang et al. 2018). Primers were fitted with Illumina adapters for multiplexing, using the Nextera XT Index Kit v2 (Illumina, Germany), and PCR reactions were performed in a Mastercycler 5341 (Eppendorf, Germany). Finally, paired‐end sequencing of 2 × 300 bp was performed using a MiSeq Reagent kit v3 and 30% Phiχ on an Illumina MiSeq platform (Illumina Inc., San Diego, CA, United States) at the Sequencing Service at Ludwig‐Maximilian University of Munich.

### 2.4 Bioinformatics Workflow

Raw reads from the MiSeq platform were demultiplexed and trimmed using default settings of the Illumina MiSeq software. The DADA2 workflow (Version1.16.0) (Callahan et al. 2016) was used in R v. 4.2.1 (R Core Team 2022) to process the resulting fastq files. Afterward, all samples below 5000 raw reads were discarded, resulting in 272 samples. In the following workflow, the raw reads were quality filtered, dereplicated, and trimmed according to standard protocols. Forward reads were trimmed after 240 bp and reverse reads after 160 bp. The resulting forward and reverse reads were merged using the DADA2 default overlap of 12 base pairs. Subsequently, chimeric sequences were removed. Taxonomic assignment was conducted using the Unite database (Version 8.3) (Abarenkov et al. 2024) and the naive Bayesian classifier method (Wang et al. 2007) implemented in DADA2. Trophic modes, guilds, and growth form were assigned using the package FUNGuildR and the FUNGuild database (Version 1.1) (Nguyen, Williams, et al. 2016). Afterward, the FUNGuild assignment for guilds was simplified by only using the terms endophyte, epiphyte, lichenized, mycorrhizal, mycoparasite, pathogen, saprotroph, symbiont, as well as combinations of these terms. Categories consisting of more than two different guilds were collected under the name “multiple lifestyles”. In a final step all non‐fungal taxa were removed. A total of 3,748,697 reads were assigned to 1573 fungal ITS1 amplicon sequence variants (ASVs) (Callahan et al. 2017).

### 2.5 Statistical Analyses

All statistical analyses were carried out using R v. 4.2.1 (R Core Team 2022); data wrangling and visualization was done with the functions provided in *tidyverse* (Wickham et al. 2019). As both the value of functional traits and their variation within a tree individual (i.e., intra‐individual variability) can have an effect on the interactions of plants with other guilds (Herrera 2017), we calculated the mean and the variance for all predicted leaf traits in every tree (Le Bagousse‐Pinguet et al. 2017) (Figure S1). Specifically, the mean value reflects the tree's strategy in terms of resource use, with leaves on the conservative side of the LES displaying higher values in LDMC and C, and those on the acquisitive side being associated with high values in SLA, N, and P (I. J. Wright et al. 2004). In contrast, the variance quantifies the degree to which trait values deviate from the mean within the canopy.

To describe alpha‐diversity of all fungi, we calculated the Abundance‐based Coverage Estimator of species richness (ACE) (Chao and Lee 1992) for each tree, using the “estimate_richness” function in *phyloseq* (McMurdie and Holmes 2013) across taxonomic levels, that is, phylum, class, order, genus, species, and additionally by trophic mode, guild, and growth form. The use of ASVs allows for the calculation of richness for a single fungal species, as these species can be composed of a variety of different ASVs (Callahan et al. 2017). ASVs of unidentified taxa on the phylum level were omitted, whereas fungi without certain taxonomic placement (Incertae sedis) were included as an own group. Fungi belonging to this group have been recognized as fungi during taxonomic assignment but could not be placed into a certain phylum with certainty, as such they could belong to all known or unknown phyla and represent an artificially merged group.

To test for the interaction of endophytic fungi with mean leaf traits (H1), we used tree species identity as random and conditional factor in uni‐ and multivariate analyses, respectively, to account for the potential host specific effects on endophytic fungi. We employed linear mixed effects models to relate the ACE of all taxonomic levels at the levels of individual trees to the mean trait values SLA, LDMC, leaf carbon, nitrogen, and phosphorus content, as well as their respective ratios (C:N, C:P; N:P) in separate single predictor models. The random intercept model incorporated tree species identity as well as TSQ nested in plot as random factors. All models were also calculated as random slope models, with additionally including the interaction of tree species with the respective trait as additional random factor. We compared the performance of random slope with random intercept models using Akaike information criterion (AIC). Since random intercept models performed generally better than random slope models, we only present results from random intercept models in the following. The results were then interpreted on the basis of a type III ANOVA to test each main effect after accounting for all other terms, including the interactions.

To assess the relationship between mean trait values and endophyte community composition, we calculated *β*‐diversity using Bray–Curtis dissimilarity, which captures differences in community composition on the basis of relative abundance. We then applied a PERMANOVA using the “*adonis2*” function in *vegan* (Oksanen et al. 2025) to test for effects of tree‐level mean trait values on endophyte community composition. Host tree species was included as a conditional variable using the “condition()” function in the model formula, which removed all variation of tree identity effects. The remaining variation was then related to the constraining variable, which were the leaf traits. Although this approach underestimates the role of leaf traits because differences in mean leaf traits between species are no longer considered, it makes sure that encountered differences are not affected in any way by the tree species' identity. To visualize the multivariate relationships between traits and endophyte communities, we used distance‐based redundancy analysis (dbRDA) implemented via the “capscale” function, also using Bray–Curtis dissimilarity.

To test whether intra‐individual trait variation in leaf traits within the same tree positively influences endophyte taxon richness (H2), we employed the exact same methodology as for H1, but used intra‐individual trait variance instead of mean trait values as a predictor.

To test whether niche differentiation is more pronounced at lower taxonomic levels of fungal endophytes than at higher levels (H3), we analyzed the model slopes from the linear mixed effects models used for testing H1 and H2. Specifically, we examined whether the direction and explanatory power of individual trait effects exhibited a phylogenetic signal across different taxonomic levels. This approach allowed us to assess the extent to which habitat specialization is conserved at various taxonomic levels. To quantify the phylogenetic signal, we calculated Blomberg's *K* (Blomberg et al. 2003) using the “phylosig” function in the *phytools* package (Revell 2024).

A phylogenetic tree was constructed from non‐chimeric ASVs using the *DECIPHER* (Wright 2016) and *phangorn* (Schliep 2011) packages. Sequences were aligned with “AlignSeqs”, and an initial tree was built using the neighbor‐joining method. A maximum likelihood tree was then inferred using “pml” and optimized with “optim.pml”, which refines model parameters such as substitution rates, gamma‐distributed rate variation, and the proportion of invariant sites. For analyses the phylogenetic tree was pruned to different taxonomic levels (class, order, family, genus, and species) using the ´tax_glom´ function in *phyloseq*. Each tree was pruned to match the taxa included in the trait model outputs for the corresponding level.

Although Blomberg's K was originally developed for use with complete phylogenies, it is methodologically valid to assess phylogenetic signals within subtrees or higher‐level clades. This approach has been widely used to investigate how the strength and nature of phylogenetic signal varies across phylogenetic scales (e.g., Münkemüller et al. 2012; Kamilar and Cooper 2013). Importantly, this does not involve re‐measuring traits, as the trait values can be averaged for the different nodes, but rather evaluates how trait conservatism shifts under different phylogenetic contexts. This allows, for example, to test if more closely related taxa within a clade exhibit a stronger or weaker signal than expected under Brownian motion.

As an additional approach for testing the phylogenetic signal, we tested for phylogenetic autocorrelation, using Moran's I (e.g., Pavoine et al. 2010; Hardy and Pavoine 2012). Moran's *I* was calculated using the same approach as above for Blomberg's K by plotting the slopes of the linear model relating ACE to single mean traits on the phylogenetic tree at each taxonomic level. Afterward, we calculated Moran's I using the “Moran. I” function without neighborhood weights in the *ape* package (Paradis and Schliep 2019). We repeated the analyses of Blomberg's *K* and Moran's *I* using mean leaf trait values instead of model slopes by calculating mean trait values per fungal taxon at the different taxonomic levels, from class down to ASV. This approach was feasible at the ASV level because it did not require computing a diversity index or fitting a corresponding model. ASVs represent distinct DNA sequences from the same genetic locus and can belong to the same species (Callahan et al. 2017). This fine‐scale resolution is valuable, as different strains within a single species may have distinct ecological niches or exhibit varying functional capacities (Constantin et al. 2021).

This study was subjected to multiple considerations regarding *p*‐value adjustment and beta error avoidance. We performed multiple statistical tests to assess the interaction of leaf traits and fungal endophyte communities at different taxonomic levels. Although *p*‐value adjustments are commonly used to control for false positives (type I errors) in multiple comparisons, we deliberately chose not to apply such corrections. Our primary goal was to identify taxa that are potentially sensitive to leaf traits, and adjusting for multiple comparisons would increase the risk of Type II errors (beta failures)—failing to detect true effects.

Given the ecological context of our study, where effect sizes can be small and biological significance is as important as statistical significance, we chose the conventional threshold of *α* = 0.05. This approach ensures that potentially meaningful ecological patterns are not missed because of overly conservative statistical adjustments. Although we acknowledge the trade‐off between Type I and Type II errors, we prioritized reducing the likelihood of missing true associations that could contribute to our understanding of microbial–plant interactions.

Our analyses use models in which leaf traits are treated as predictors of fungal community composition. We acknowledge that these relationships are correlational, as leaf traits were not directly manipulated. However, we had to build our models in this way for several reasons. First, our main focus was on leaf endophytes, that is, to describe which factors affect leaf endophyte communities. Thus, the endophytes were always the responses in our model. Second, the models had to take into account random factors, such as tree species identity and spatial configuration, to predict endophyte responses. This also requires defining a direction of the effects. This does not preclude that random factors might also affect leaf traits.

## 3 Results

All significant correlations of leaf traits with endophyte richness were encountered only for the phyla Ascomycota and Basidiomycota, whereas the phyla Chytridiomycota and Mortierellomycota did not yield any significant relationships, because of their low relative abundance, which together accounted for fewer than 0.5% of total reads. Within the Ascomycota and Basidiomycota, all significant relationships were encountered in two ascomycete classes, Dothideomycetes and Eurotiomycetes, and three basidiomycete classes, Agaricostilbomycetes, Cystobasidiomycetes, and Tremellomycetes (Table S2). Interactions of leaf traits and endophytes were measurable at all taxonomic levels ranging from phylum to species.

### 3.1 Relationship of Endophytes and Mean Trait Values

**FIGURE 1:**
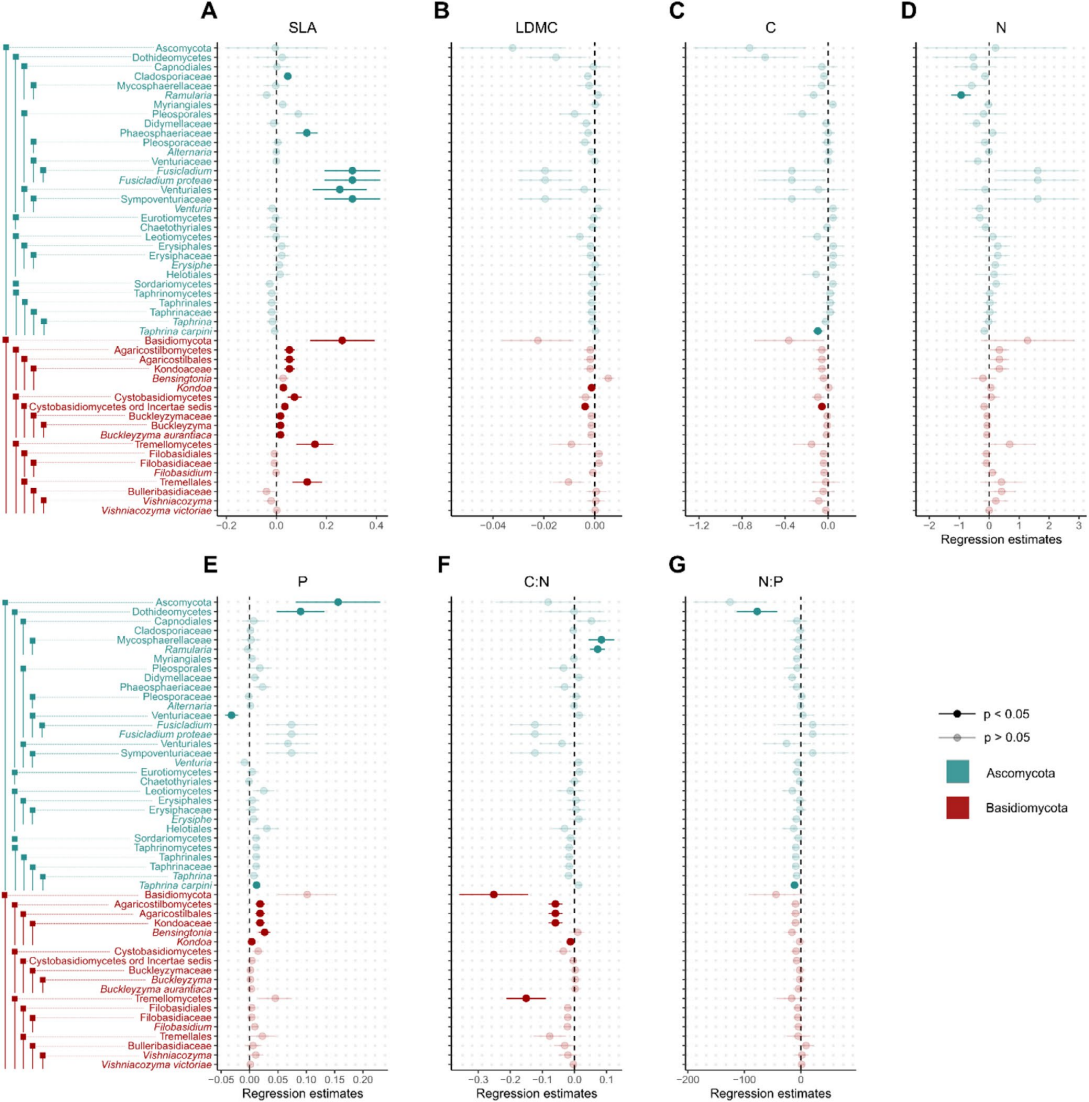
Regression parameter estimates of the linear mixed effects models assessing the abundance‐based Coverage Estimator (ACE) of endophytic taxa as a function of mean trait values, including error bars showing standard errors, are presented for the following traits: Traits (A) specific leaf area (SLA), (B) leaf dry matter content (LDMC), (C) leaf C, (D) leaf N, (E) leaf P, (F) leaf C:N ratio, and (G) leaf N:P ratio. Significant relationships are highlighted with bold symbols. Panels are arranged by the number of significant relationships. For model results, see Table S2. Ascomycota are shown in blue, whereas Basidiomycota are shown red. For a version of this figure showing guild and trophic mode form, see Appendix Figure S2.

Among the examined leaf traits, SLA emerged as the strongest predictor of endophyte richness, influencing 28 out of 83 significant interactions between endophytic taxa and mean leaf traits. It increased the relative abundances of the phylum Basidiomycetes as well as the basidiomycete classes Agaricostilbomycetes, Cystobasidiomycetes, and Tremellomycetes (Figure 1A; Table S2). Additionally, the richness of several orders, families, genera, and species of Agaricostilbomycetes, Cystobasidiomycetes, Dothideomycetes, and Tremellomycetes was positively related to SLA (Figure 1A; Table S2). Furthermore, SLA increased the richness of several guilds (pathogen, saprotroph, epiphyte‐saprotroph, and fungal parasite‐saprotroph), growth forms (dimorphic fungi, dimorphic facultative yeasts, and tremelloid yeasts), and trophic modes (pathotroph, saprotroph, and sapro‐symbiotroph) (Figure S2; Table S3).

Exploring the relationship with leaf chemical traits, we found leaf phosphorus content to be the most influential factor. Phosphorus increased the richness of the phylum Ascomycota, including the class of Dothideomycetes, as well as the Basidiomycete class Agaricostilbomycetes, including the order Agaricostilbales, the family Kondaeceae, and the genera *Bensingtonia* and *Kondoa* (Figure 1E; Table S2) and dimorphic fungi (Table S3). The leaf C:N ratio had a negative relationship with overall Basidiomycota richness as well as the basidiomycete class Agaricostilbomycetes, including the order Agaricostilbales, the family Kondaeceae, and the genus *Kondoa* (Figure 1F; Table S2) and saprotrophic and dimorphic fungi (Table S3). Further, we found C:N to positively affect ascomycete family Mycosphaerellaceae and genus *Ramularia* (Dothideomycetes).

LDMC displayed a consistently negative relationship with endophyte richness, as for the basidiomycete class Cystobasidiomycetes Incertae sedis and the genus *Kondoa* (Agaricostilbomycetes) (Figure 1B; Table S2), as well as the guild of pathogens, and the trophic modes of pathotrophs and sapro‐symbiotrophs were negatively influenced by LDMC (Figure S2; Table S3).

Furthermore, we found leaf carbon to decrease the richness of Cystobasidiomycetes Incertae sedis and *Taphrina carpini* (Figure 1C; Table S2). Similarly, ASV richness of the class Dothideomycetes and *Taphrina carpini* (a taxon composed of 10 ASVs in our study) was negatively influenced by the leaf N:P ratio (Figure 1G; Table S2), whereas leaf nitrogen content decreased the richness of the genus *Ramularia* (Dothideomycetes) (Figure 1D; Table S2). The N:P ratio exhibited no significant relationships with endophyte richness.

### 3.2 Relationship of Endophytes and Intra‐Individual Trait Variation

**FIGURE 2:**
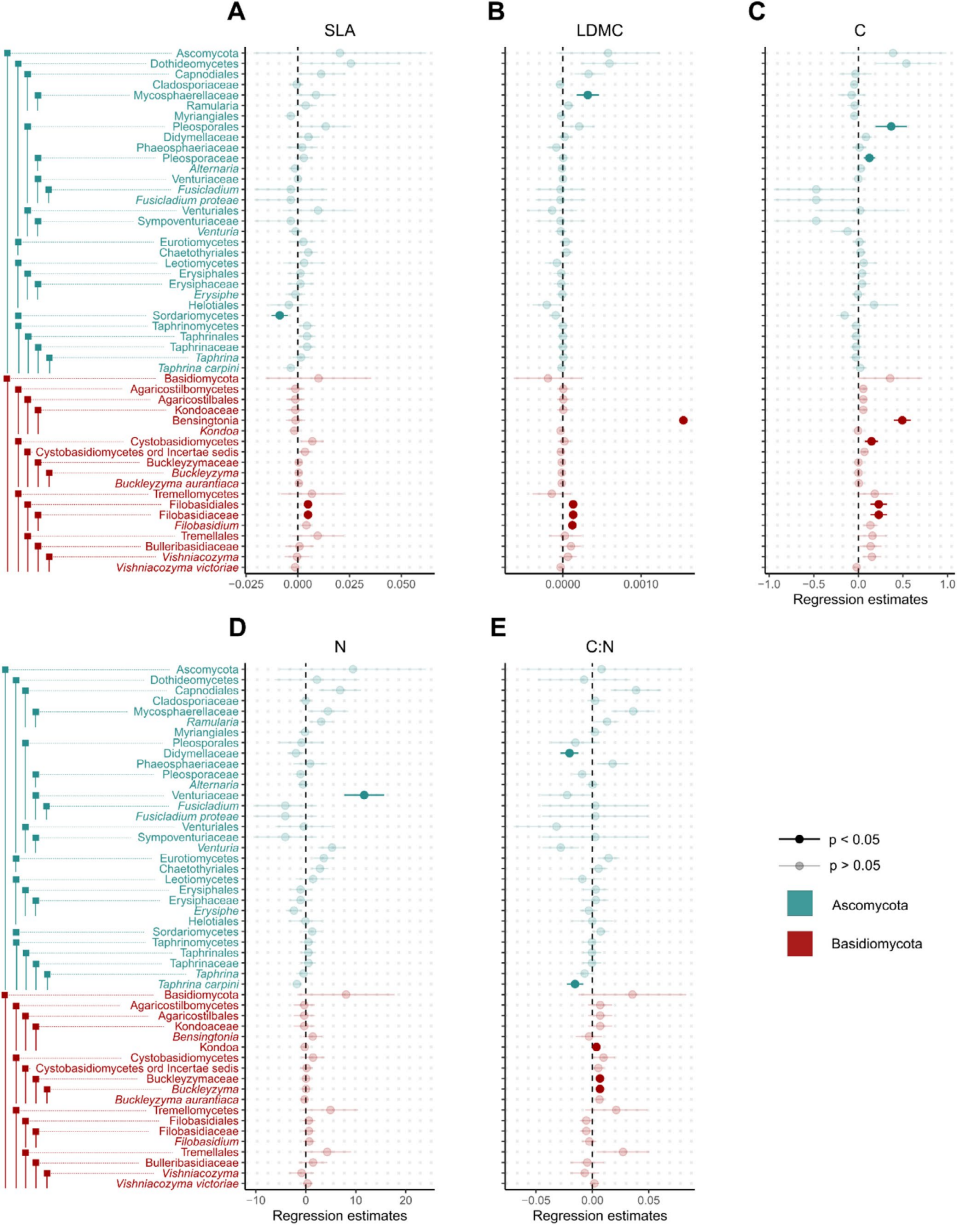
Regression parameter estimates of the linear mixed effects models assessing the abundance‐based Coverage Estimator (ACE) of endophytic taxa as a function of intra‐individual trait variance, including error bars showing standard errors, are presented for the following traits: Traits (A) specific leaf area (SLA), (B) leaf dry matter content (LDMC), (C) leaf C, (D) leaf N, (E) leaf P, (F) leaf C:N ratio, and (G) leaf N:P ratio. Significant relationships are highlighted with bold symbols. Panels are arranged by the number of significant relationships. For model results see Table S2. Ascomycota are shown in blue, whereas Basidiomycota are shown in red. For a version of this figure showing guild and trophic mode form, see Appendix Figure S3.

Analyzing the relationship of endophyte richness with intra‐individual trait variation, we found mainly positive relationships (Figure 2; Table S2). We found intra‐individual leaf carbon variation to affect multiple ascomycete and basidiomycete taxa as well as the growth form of dimorphic facultative yeasts (Figure 2C; Table S2, Table S3). Taxa that responded positively to leaf carbon variation included the class of Cystobasidiomycetes, the order Pleosporales (Dothideomycetes), the family Pleosporaceae (Dothideomycetes), the order Filobasidiales (Tremellomycetes), the family Filobasidiaceae (Tremellomycetes), and the genus *Bensingtonia* (Tremellomycetes) (Figure 2C; Table S2). Further, we found the variation in the leaf C:N ratio to positively influence the family Buckleyzymaceae, the genus *Buckleyzyma*, and the genus *Kondoa*, all belonging to the Basidiomycota (Figure 2E; Table S2). Additionally, the guilds of fungal parasites and epiphyte‐saprotrophs were reported to positively react to the variation in the leaf C:N ratio (Figure S3; Table S3). Furthermore, variation of the C:N ratio decreased the richness of the family Didymellaceae and *Taphrina carpini*, both belonging to the Ascomycota (Figure 2E; Table S2).

Both the variation in LDMC and SLA positively affected endophyte richness of the order Filobasidiales (Tremellomycetes), the family Filobasidiaceae (Tremellomycetes), and in case of LDMC also on the genus *Filobasidium* (Tremellomycetes) (Figure 2A,B; Table S2). Additionally, LDMC increased the richness of the guild of patho‐saprotrophs, whereas SLA increased the richness of fungi growing as yeasts (Figure S3; Table S3). Intra‐individual leaf nitrogen variation positively influenced the richness in the family Venturiaceae (Dothideomycetes) (Figure 2D; Table S2).

Overall, we encountered only three negative interactions of intra‐individual trait variation with endophyte richness; these were those of the C:N ratio on Didymellaceae (Dothideomycetes) and *Taphrina carpini* and of SLA on the richness of Sordariomycetes. Variation of leaf phosphorus content did not significantly affect the richness of endophytes (Table S2).

### 3.3 Endophyte Community Composition

**FIGURE 3:**
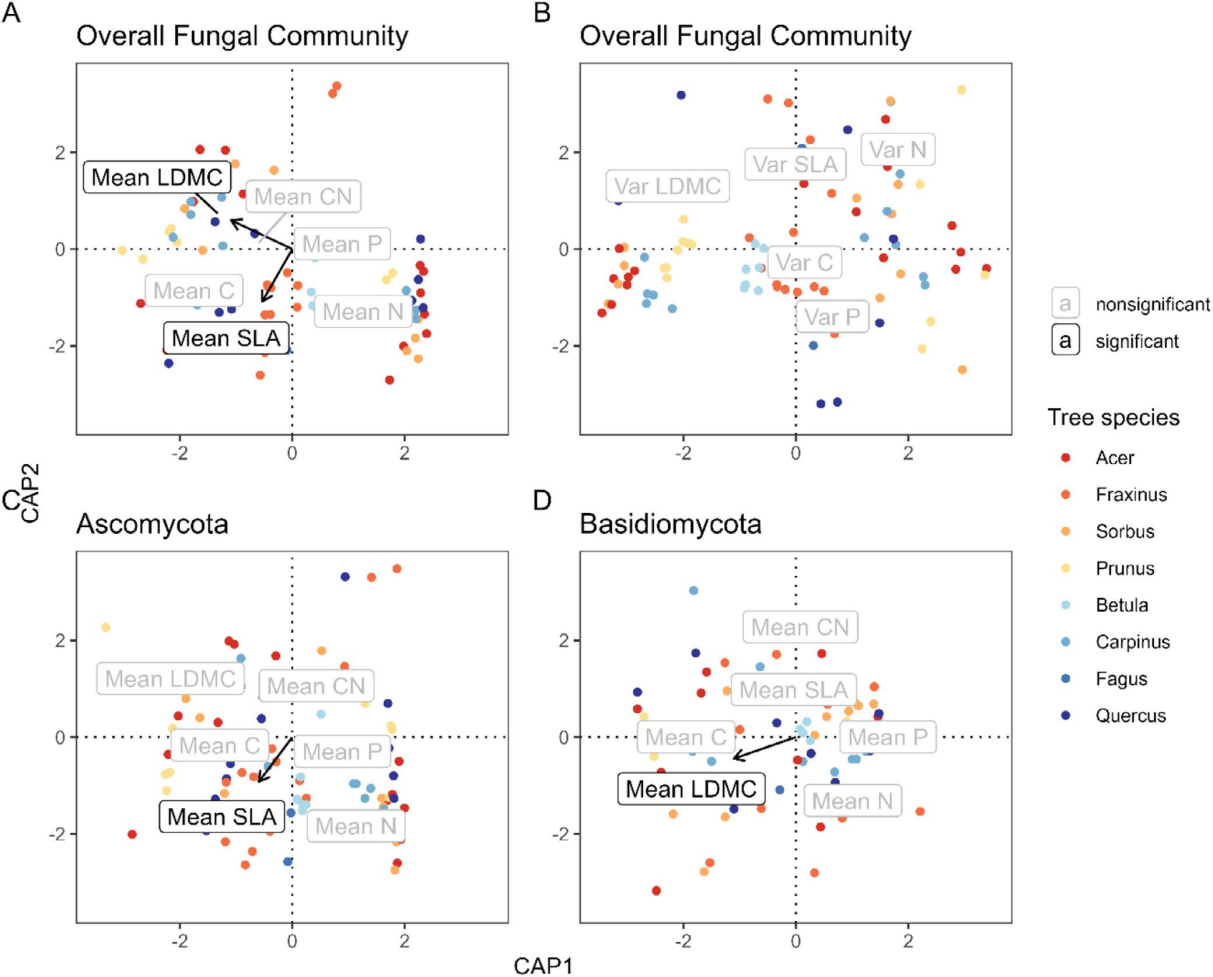
Distance‐based redundancy analysis (dbRDA) of the (A, B) overall fungal community composition as well as the phyla (C) Ascomycota or (D) Basidiomycota using tree species and (A, C, D) mean trait values or (B) intra‐individual trait variation as constraining variable. Overall fungal community composition was affected by (A) mean trait values of SLA (*p* = 0.047) and LDMC (*p* = 0.033) (Table S4) according to a permutation test, whereas for (B) intra‐individual trait variation, no significance was encountered (Table S5). The permutation tests for the individual phyla revealed mean trait values of SLA (*p* = 0.037) to significantly affect (C) Ascomycota, whereas (D) Basidiomycota were significantly influenced by mean values of LDMC (*p* = 0.019) (Table S4). Arrows and labels of traits showing significant correlations are shown in bold. Red, orange, and yellow colors represent AM associated tree species and blue colors EM associated tree species.

In the previous linear models, we used species identity as a random factor. In the multivariate models, we had to completely remove species identity effects. We did this by removing their effect as conditional factors. This also removed effects that were jointly explained by species identity and traits, leaving only effects exclusively brought about by traits. We found significant relationships between overall endophyte community composition and both SLA and LDMC. PERMANOVA outputs revealed 1.2% of total variance explained by each of these traits, whereas host species identity explained a total variance of 32% (Figure 3A; Table S4). Testing the relationship between mean trait values and individual phyla revealed a significant relationship of Ascomycota with SLA (Figure 3C; Table S4), whereas Basidiomycota showed a significant relationship with LDMC (Figure 3D; Table S4). Furthermore, we tested the effect of mean trait values on the community composition of the four most prevalent endophyte classes and found the ascomycete class Leotiomycetes to be significantly influenced by LDMC, whereas all other classes (Dothideomycetes, Taphrinomycetes, and Tremellomycetes) showed no significant relationships (Table S4).

We repeated the analyses testing for a relationship of intra‐individual trait variation and fungal endophyte community composition. The overall fungal community composition showed no relationship with intra‐individual trait variation, neither for the phyla Ascomycota and Basidiomycota, nor for the most prevalent classes (Figure 3B; Table S5).

### 3.4 Effect of Taxonomic Resolution

**FIGURE 4:**
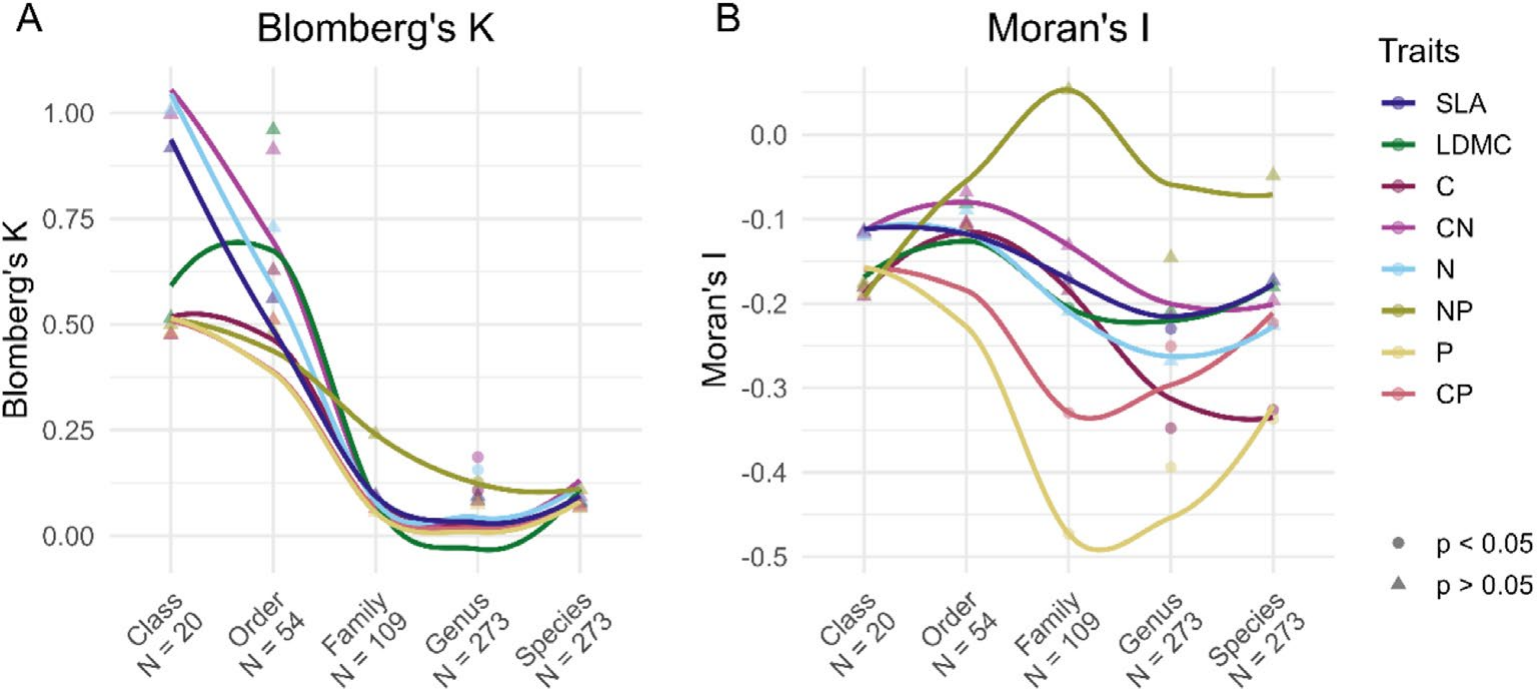
Local regression plots of (A) Blomberg's *K* and (B) Moran's *I* for model slopes from the linear mixed effects models of mean trait values (Figure 1) of the specific leaf area (SLA), leaf dry matter content (LDMC), leaf C, leaf C:N ratio, leaf N, leaf N:P ratio, leaf P, and leaf C:P ratio of the hosts trees of fungal endophytes, aggregated at different taxonomic levels of the endophytes. Lines were plotted using locally estimated scatterplot smoothing (LOESS). N gives the number of tips in the respective phylogeny. Significant departure from a Brownian model (Blomberg's K) or a significant autocorrelation (Moran's I) are indicated by circle shapes. For values of *K* and *I* as well as respective *p*‐values, see Table S6.

We tested if the relationships between leaf traits and endophytic fungi exhibited a phylogenetic signal and if these signals became stronger with an increasing taxonomic resolution. Using model slopes from the linear mixed effects models used for testing H1 and H2, we found all traits to adhere to a general pattern of a high value for Blomberg's *K* on the class level, followed by a sharp decline to genus level and then by an increase on the species level (Figure 4A; Table S6). Nevertheless, we could only identify significant phylogenetic signals on the genus level for leaf C (*p* = 0.001), N (*p* = 0.001), C:N ratio (*p* = 0.001), and N:P ratio (*p* = 0.001) (Table S6). The corresponding analysis testing for phylogenetic autocorrelation using Moran's *I* revealed only negative values, indicating that closely related species tend to differ more in the trait than would be expected by assigning random trait values (Figure 4B; Table S6). However, autocorrelations were only significant on the family level for LDMC (*p* = 0.044), P (*p* < 0.001), and C:P ratio (*p* < 0.001), on the genus level for SLA (*p* = 0.019), C (*p* = 0.01), P (*p* < 0.001), and C:P (*p* = 0.004), and on the species level for C (*p* = 0.013), P (*p* = 0.002), and C:P ratio (*p* = 0.007) (Figure 4B, Table S6).

Using mean trait values and the endophytes phylogeny, we found Blomberg's *K* to gradually decrease from class to species level (Figure S4A; Table S7). Here, significant phylogenetic signals were detected on the family level for LDMC (*p* = 0.001), C (*p* = 0.047), N (*p* = 0.009), C:N ratio (*p* = 0.007), and C:P ratio (*p* = 0.018) (Table S7). Furthermore we found N (*p* = 0.001) and C:N ratio (*p* = 0.047) to be significant on the genus level, whereas N (*p* = 0.001) and C:N (*p* = 0.049) were shown to be significant on the species level and C:P (*p* = 0.028) on the ASV level.

Subjecting the mean leaf values to the test on phylogenetic autocorrelation using Moran's *I* also mainly showed negative, albeit insignificant, values (Figure S4B; Table S7).

## 4 Discussion

By analyzing fungal endophytes and traits from the same leaves, we showed that leaf traits can significantly influence leaf endophyte richness and community composition. Although the effects of leaf traits were superimposed by those of tree species identity, the relationship with leaf traits remained significant after all tree species identity effects had been removed from the models. Furthermore, our analyses revealed such relationships not only among species but also at the within‐species level and intra‐individual tree level.

Our findings support our first hypothesis that high values of traits on the acquisitive side of the LES, such as leaf phosphorus content and SLA, positively affected endophyte richness, whereas high values of traits on the conservative side of the LES, such as LDMC and carbon content exhibited negative influence toward endophyte richness. Thus, the host tree's growth strategy, as reflected by the LES, played a major role in shaping phyllosphere richness and community composition, as had been already proposed by Kembel and Mueller (2014). These results also conform to those reported by Tellez et al. (2022), who described a significant negative effect of leaf nitrogen content on endophyte richness. At the same time, the findings contradict those of Yang et al. (2016), who reported leaf carbon to exhibit a positive effect on endophyte richness. Interestingly, not all taxonomic fungal groups responded in the same way to the LES. We found that one family and one genus in the Ascomycota positively interacted with the C:N ratio, whereas all negative interactions were restricted to Basidiomycota. Such contrasting response patterns of different fungal taxon groups have rarely been reported. For example, Tellez et al. (2022) showed that the abundance of Xylariales (Sordariomycetes) and Botryosphaeriales (Dothideomycetes) responded in opposite directions to individual leaf traits.

The interplay of nutrient availability, functional leaf traits, and endophytes seems to be quite intricate. Endophytes can increase and alter nitrogen uptake und allocation (Christian et al. 2019), but an excess supply of limiting nutrients such as nitrogen and phosphorus was reported to reduce endophyte richness (Rasmussen et al. 2007; Meng et al. 2022). However, in our study, we encountered only limited evidence for such interactions, which could have resulted from only including samples from trees that grew under similar soil conditions. As leaf P content was primarily associated with increased endophyte richness, whereas the N:P ratio showed a negative relationship, we would assume that inhibitive effects on endophytes are mainly caused by high leaf N content. This interpretation is supported by the findings of Tellez et al. (2022) of negative relationship between leaf nitrogen content on endophyte richness.

Previously, it had been proposed that a higher intra‐individual variation could attract a more diverse community of consumers (Herrera 2017). Our findings allow expanding this idea to endophytes, as intra‐individual leaf variation exhibited a mostly positive relationship with leaf endophyte richness, thus confirming our second hypothesis. One of the most striking results was that leaf carbon content variation affected endophyte richness, even though leaf carbon showed very little variation in itself. Further, we found a positive interaction with LDMC variation, whereas higher LDMC mean values had often been reported to negatively influence endophytes richness (Tellez et al. 2022).

A possible explanation for the positive interaction between intra‐individual trait variation and foliar fungal endophytes is the creation of more different niche opportunities because of more heterogeneous leaf microsites within the tree crown. This heterogeneity is likely brought about by structural and spatial differences within the canopy, where a range of light intensities and humidity levels meet and simultaneously shape leaves with different levels of trait expression (Proß et al. 2021). Additionally, it has been proposed that intra‐individual trait variation can enhance ecosystem functioning (Sobral 2023; Proß et al. 2024). Although we did not explicitly measure ecosystem functions, studies showing the positive effect of endophytes on plant growth (Arnold et al. 2003; König et al. 2018; Christian et al. 2019), together with our results on the interaction between intra‐individual trait variation and endophyte richness, suggest an indirect positive effect of intra‐individual trait variation on ecosystem functioning via fungal endophytes. In consequence, intra‐individual trait variation should be considered a predictor for future studies on fungal endophytes, as it may help to explain previously inconclusive results on endophyte–host trait relationships.

Although endophyte richness was influenced by mean values and intra‐individual variation of several leaf traits, leaf fungal community composition responded mainly to mean values of SLA and LDMC. This is likely due to strong host species identity effects. As different tree species differ in their leaf trait spaces, this should also differ in their microbiomes (Kambach et al. 2021). Interestingly, dividing the fungal community by phylum revealed that the Ascomycota community composition was only influenced by SLA, whereas the Basidiomycota community composition was mainly structured by LDMC. Although this interaction with LDMC had been reported previously (Kembel and Mueller 2014; Tellez et al. 2022), to our knowledge no such interactions with SLA have been described so far. Previous studies assumed that endophyte communities were directly influenced by vertical gradients of light and water availability in the crown (Wang et al. 2024). Our results suggest that endophytes are mainly influenced indirectly by these gradients via leaf traits, accompanied by a complex interaction of fungal taxa directly affected by the changing abiotic conditions.

Our results on Blomberg's K suggest that the strength of phylogenetic signals in both model slopes and mean traits decreases with the increasingly lower taxonomic level (i.e., family and genus), suggesting that some aspects of habitat specificity are conserved across deep evolutionary lineages. Thus, the expectation from the Brownian motion model for endophyte niche evolution only applies to higher taxonomic levels (i.e., class and order). This aligns with our third hypothesis that niche preferences may have originated early in fungal evolution. This pattern supports the idea that broader clades retain more consistent ecological characteristics, whereas divergence occurs as lineages diversify. However, we have to take into consideration that the tests at the higher taxonomic levels were insignificant, probably because of a low number of nodes at these levels. In contrast, Moran's *I* revealed mostly negative and not positive autocorrelations, also indicating that closely related species responded differently to the leaf traits. We found Blomberg's *K* to be more sensitive at high taxonomic levels. Overall, these results suggest that endophyte leaf trait niches carry a legacy of early evolutionary history, whereas more recent evolutionary processes have driven niche differentiation with respect to host tree traits. This niche partitioning likely promotes the coexistence of closely related fungal species by reducing direct competition.

Strong phylogenetic signals of leaf traits for plant communities have been reported multiple times (Chang and HilleRisLambers 2019; Meireles et al. 2020; Ávila‐Lovera et al. 2023). Leaf traits associated with water relations, such as leaf area, stomatal density, and vein density, which influence how plants acquire, utilize, and conserve water, tend to be more similar among closely related plant species (Ávila‐Lovera et al. 2023). A possible explanation for the effect of leaf traits on endophytic fungi could be that leaf reflectance spectra, which encapsulate multiple chemical and morphological characteristics of leaves, and therefore characterize endophytes potential niches, exhibit strong phylogenetic signals across various levels of the plant phylogenetic tree, such as order and family (Meireles et al. 2020). The reflectance spectrum of leaves can be used to describe the overall chemical and morphological composition of leaves (Kokaly et al. 2009). As leaf traits show phylogenetic signals throughout the plant kingdom and define the habitat of fungal endophytes, it is reasonable to assume that these phylogenetic signals are also translated into the fungal endophyte community. Additionally, the close link of leaf traits and fungal endophytes is in accordance with previously reported co‐evolution of fungal pathogens and their host plants (Rutten et al. 2021). It is likely that the strong phylogenetic signals at family and genus level are a result of a long past co‐evolution, potentially driven by environmental conditions that filtered both the local endophyte species pool and leaf trait values. To further investigate these questions, it would be necessary to study a broader range of host plants.

## 5 Conclusion

This study reveals that intra‐individual variation in leaf traits plays a key role in shaping foliar fungal endophyte communities, influencing both richness and composition. Specific traits such as SLA and LDMC have distinct and contrasting effects on community structure, underscoring the importance of trait heterogeneity within individual trees. Although leaf traits shape fungal communities, our findings also suggest potential feedbacks through microbe‐plant interactions. By proposing a testable, trait‐based framework for understanding endophyte assembly, this work lays the foundation for future research across ecosystems and taxa, with experimental validation as an important next step.

## Author Contributions

**Michael Köhler:** conceptualization (lead), data curation (equal), formal analysis (lead), visualization (equal), writing – original draft (lead), writing – review and editing (lead). **Pablo Castro Sánchez‐Bermejo:** data curation (equal), formal analysis (equal), visualization (equal), writing – review and editing (equal). **Georg Hähn:** formal analysis (equal), visualization (equal), writing – review and editing (equal). **Olga Ferlian:** writing – review and editing (equal). **Nico Eisenhauer:** funding acquisition (equal), writing – review and editing (equal). **Tesfaye Wubet:** funding acquisition (equal), writing – review and editing (equal). **Sylvia Haider:** funding acquisition (equal), writing – review and editing (equal). **Helge Bruelheide:** formal analysis (equal), funding acquisition (equal), project administration (lead), resources (lead), supervision (lead), writing – review and editing (equal).

## Conflicts of Interest

The authors declare no conflicts of interest.

## Supporting information


Data S1.

## Data Availability

The amplicon data generated for this study can be found in the Sequence Read Archive (SRA) of the National Centre for Biotechnology Information (NCBI) under bioproject number PRJNA1120618. The data used in this study is publicly available at the MyDiv Experiment data portal: doi.org/10.25829/2QXM‐DK15. The code used in this study is publicly available at: https://doi.org/10.5281/zenodo.15056014.
