# Supplementary material for: Foliar Endophytic Fungal Communities Are Driven by Leaf Traits—Evidence From a Temperate Tree Diversity Experiment
Source: Ecol Evol. 2025 Jul 17;15(7):e71691. doi: 10.1002/ece3.71691 (PMC12268234; doi:10.1002/ece3.71691)
Supplement: Supplementary file 1 — Data S1. [file ECE3-15-e71691-s001.docx]

Appendix

**Foliar endophytic fungal communities are driven by leaf traits - evidence from a temperate tree diversity experiment**

Michael Köhler ^1, 2^, Pablo Castro Sánchez-Bermejo ^1, 2, 3^, Georg Hähn ^4^, Olga Ferlian ^2, 5^, Nico Eisenhauer ^2, 5^, Tesfaye Wubet ^6, 2^, Sylvia Haider ^3^, Helge Bruelheide ^1, 2^

Manuscript correspondence: Michael.koehler@botanik.uni-halle.de


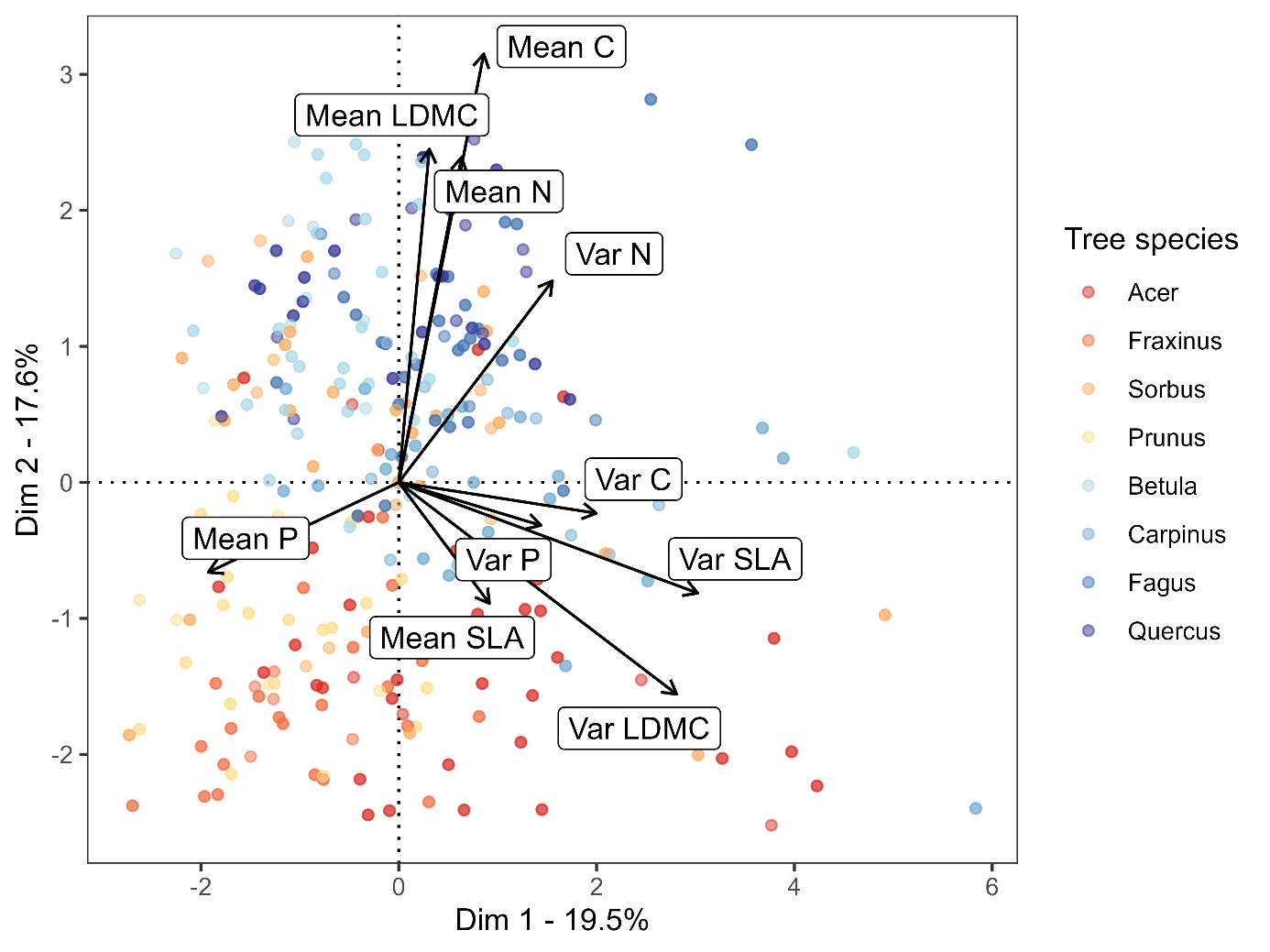


Fig. S1: Principal component analysis (PCA) of the calculated mean value and variance for specific leaf area (SLA), leaf dry matter content (LDMC), leaf C, leaf N, leaf P, leaf in every tree. Red, orange and yellow colors represent AM associated tree species, while blue colors represent EM associated tree species.


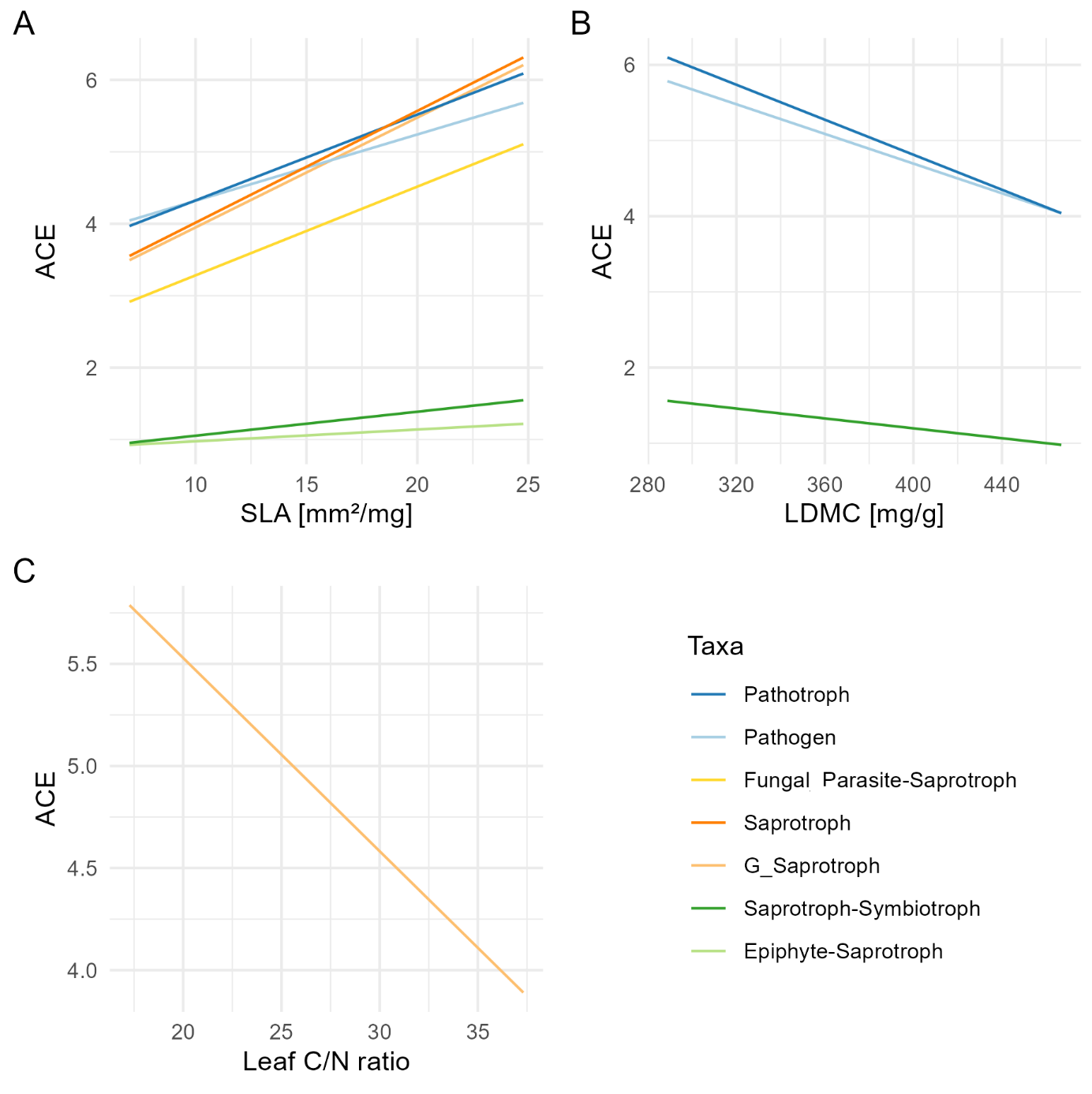


Fig. S2: Abundance-based Coverage Estimator (ACE) of fungal trophic modes (Pathotroph, Saprotroph, Saprotroph-Symbiotroph) and guilds (Pathogen, Fungal Parasite-Saprotroph, Saprotroph [“G_Saprotroph”], Epiphyte-Saprotroph) as function of mean trait values of (A) SLA, (B) LDMC, and (C) leaf CN ratio. Displayed are only significant relationships according to linear mixed effects models for the combination of taxa and trait mean value, with species composition, plot, TSQ and tree species as random factors. For model results see Table S3.


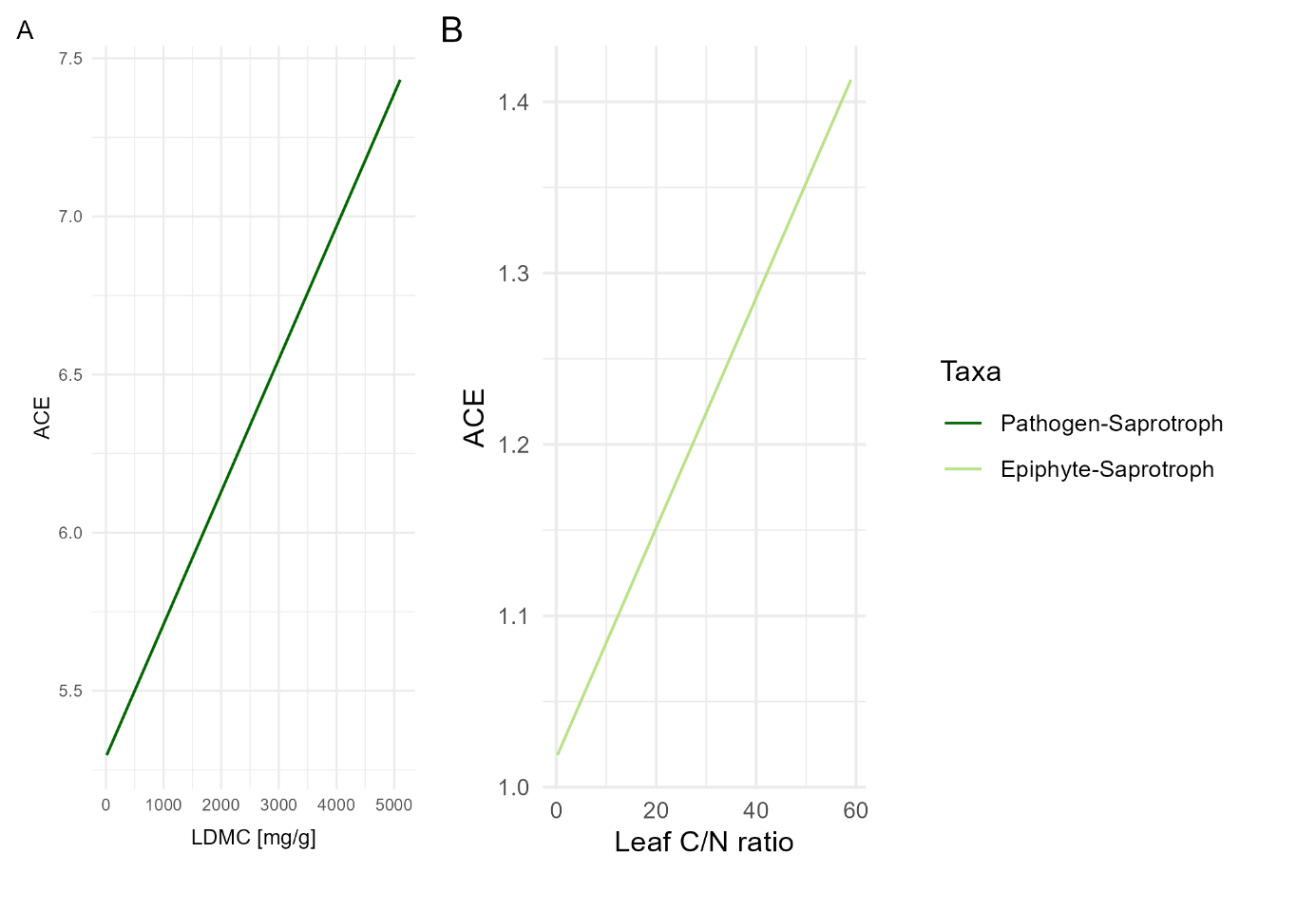


Fig. S3: Abundance-based Coverage Estimator (ACE) of fungal guilds as function of intraindividual trait variation of (A) LDMC and (B) leaf CN ratio. Displayed are only significant relationships according to linear mixed effects models for the combination of taxa and trait mean value, with species composition, plot, TSQ and tree species as random factors. For model results see able S3.


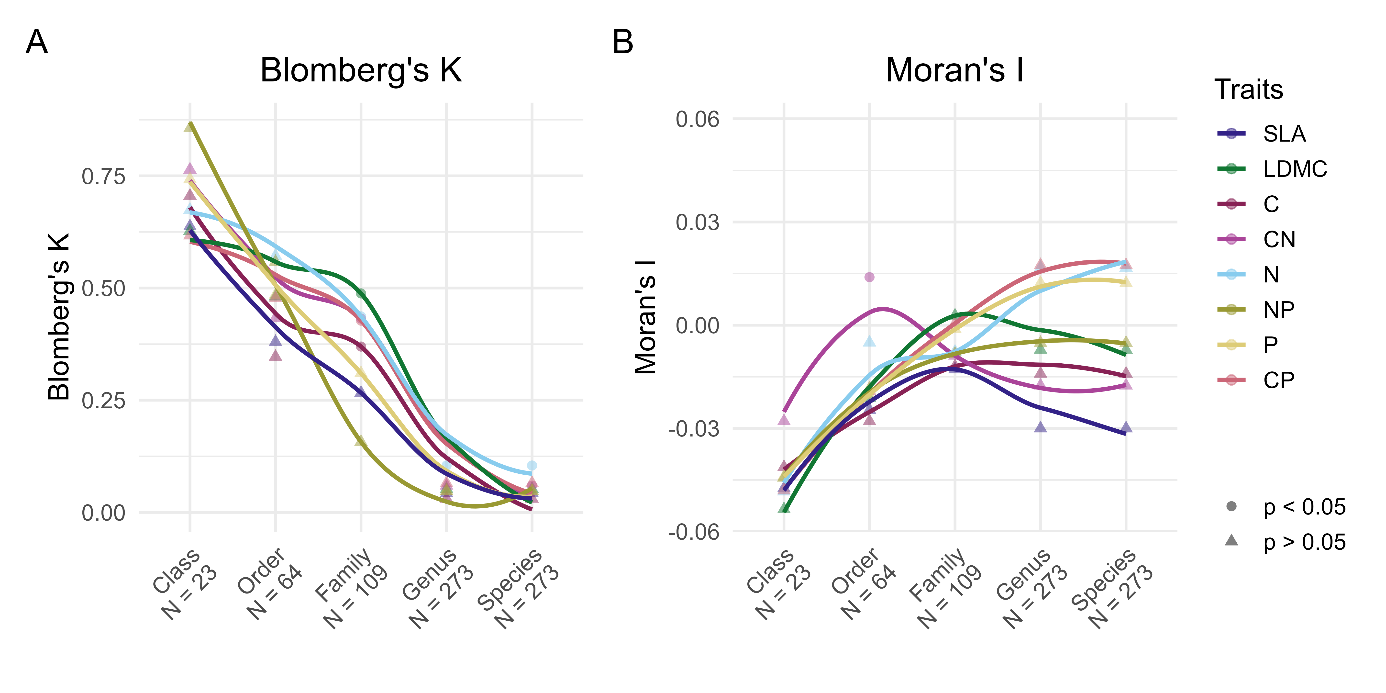


Fig. S4: Local regression plots of (A) Blomberg’s K and (B) Moran’s I for phylogenetic signal in host leaf trait values of specific leaf area (SLA), leaf dry matter content (LDMC), leaf C, leaf C:N ratio, leaf N, leaf N:P ratio, leaf P, and leaf C:P ratio, calculated as mean trait value per fungal taxon at the different taxonomic levels. Lines were plotted using locally estimated scatterplot smoothing (LOESS). N gives the number of tips in the respective phylogeny. Significant departure from a Brownian model (Blomberg’s K) or a significant autocorrelation (Moran’s I) are indicated by circle shapes. For values of K and I as well as respective p-values see Table S7.

Table S1: Count of plots and samples per species combinations.

| Species combination | Plots | Samples |
| --- | --- | --- |
| Ac,-,-,- | 2 | 15 |
| Ac,Be,-,- | 1 | 8 |
| Ac,Fr,-,- | 1 | 8 |
| Ac,Fr,Pr,So | 2 | 16 |
| Ac,Pr,-,- | 1 | 8 |
| Ac,So,-,- | 1 | 8 |
| Ac,So,Ca,Qu | 1 | 3 |
| Be,-,-,- | 2 | 14 |
| Be,Ca,-,- | 1 | 8 |
| Be,Ca,Fa,Qu | 2 | 14 |
| Be,Fa,-,- | 1 | 6 |
| Be,Qu,-,- | 1 | 6 |
| Ca,-,-,- | 2 | 12 |
| Ca,Fa,-,- | 1 | 8 |
| Ca,Qu,-,- | 1 | 6 |
| Fa,-,-,- | 2 | 15 |
| Fa,Qu,-,- | 1 | 8 |
| Fr,-,-,- | 1 | 8 |
| Fr,Ca,-,- | 1 | 8 |
| Fr,Fa,-,- | 1 | 8 |
| Fr,Pr,-,- | 1 | 8 |
| Fr,So,-,- | 1 | 7 |
| Pr,-,-,- | 2 | 11 |
| Pr,Be,-,- | 1 | 8 |
| Pr,So,-,- | 1 | 8 |
| Pr,So,Be,Qu | 1 | 7 |
| Qu,-,-,- | 2 | 12 |
| So,-,-,- | 2 | 12 |
| So,Ca,-,- | 1 | 6 |
| So,Qu,-,- | 1 | 6 |

Table S2: Linear mixed effect model results for multiple types of fungal endophytic taxa, testing the effect of mean leaf trait values (Mean) and intraindividual trait variation (Var) on ACE, separately throughout taxonomic ranks ranging from phylum to species (Taxon). Shown are estimates and standard errors (se) of marginal effects of fixed factors as well as p-values and Akaike information criterion (AIC). Shown are only effects significant using ACE.

| Type | Taxon | Predictor | Estimate | SE | df | t | P | AIC |
| --- | --- | --- | --- | --- | --- | --- | --- | --- |
| Order | Agaricostilbales | Mean CN | -0.059 | 0.022 | 85.321 | -2.686 | 0.009 | 285.913 |
| Order | Agaricostilbales | Mean P | 0.019 | 0.009 | 103.228 | 2.145 | 0.034 | 296.641 |
| Order | Agaricostilbales | Mean SLA | 0.052 | 0.021 | 84.565 | 2.518 | 0.014 | 223.222 |
| Class | Agaricostilbomycetes | Mean CN | -0.059 | 0.022 | 85.321 | -2.686 | 0.009 | 285.913 |
| Class | Agaricostilbomycetes | Mean P | 0.019 | 0.009 | 103.228 | 2.145 | 0.034 | 296.641 |
| Class | Agaricostilbomycetes | Mean SLA | 0.052 | 0.021 | 84.565 | 2.518 | 0.014 | 223.222 |
| Phylum | Ascomycota | Mean P | 0.156 | 0.074 | 142.011 | 2.095 | 0.038 | 1534.085 |
| Phylum | Basidiomycota | Mean CN | -0.252 | 0.108 | 150.855 | -2.331 | 0.021 | 1321.008 |
| Phylum | Basidiomycota | Mean SLA | 0.263 | 0.129 | 134.208 | 2.045 | 0.043 | 1197.000 |
| Genus | Bensingtonia | Mean P | 0.027 | 0.010 | 28.394 | 2.576 | 0.015 | 83.422 |
| Genus | Bensingtonia | Var C | 0.492 | 0.095 | 32.330 | 5.205 | < 0.001 | 59.857 |
| Genus | Bensingtonia | Var LDMC | 0.002 | 0.000 | 25.178 | 41.784 | < 0.001 | 23.148 |
| Genus | Buckleyzyma | Mean SLA | 0.016 | 0.008 | 105.798 | 2.063 | 0.042 | 53.747 |
| Genus | Buckleyzyma | Var CN | 0.007 | 0.003 | 108.977 | 1.989 | 0.049 | 75.630 |
| Species | Buckleyzyma aurantiaca | Mean SLA | 0.016 | 0.008 | 104.811 | 2.042 | 0.044 | 54.460 |
| Family | Buckleyzymaceae | Mean SLA | 0.016 | 0.008 | 105.798 | 2.063 | 0.042 | 53.747 |
| Family | Buckleyzymaceae | Var CN | 0.007 | 0.003 | 108.977 | 1.989 | 0.049 | 75.630 |
| Family | Cladosporiaceae | Mean SLA | 0.046 | 0.013 | 44.900 | 3.428 | 0.001 | 131.498 |
| Class | Cystobasidiomycetes | Mean SLA | 0.073 | 0.028 | 88.063 | 2.587 | 0.011 | 395.390 |
| Class | Cystobasidiomycetes | Var C | 0.147 | 0.073 | 129.820 | 2.021 | 0.045 | 369.903 |
| Class | Cystobasidiomycetes Incertae sedis | Mean C | -0.057 | 0.025 | 128.000 | -2.231 | 0.027 | 185.643 |
| Class | Cystobasidiomycetes Incertae sedis | Mean LDMC | -0.004 | 0.001 | 43.917 | -3.253 | 0.002 | 199.400 |
| Class | Cystobasidiomycetes Incertae sedis | Mean SLA | 0.034 | 0.013 | 29.450 | 2.540 | 0.017 | 200.072 |
| Family | Didymellaceae | Var CN | -0.020 | 0.008 | 93.275 | -2.514 | 0.014 | 233.037 |
| Class | Dothideomycetes | Mean NP | -77.367 | 35.847 | 140.724 | -2.158 | 0.033 | 1044.423 |
| Class | Dothideomycetes | Mean P | 0.090 | 0.042 | 170.833 | 2.141 | 0.034 | 1272.840 |
| Family | Filobasidiaceae | Var C | 0.228 | 0.093 | 61.990 | 2.459 | 0.017 | 107.502 |
| Family | Filobasidiaceae | Var LDMC | 0.000 | 0.000 | 60.385 | 2.421 | 0.018 | 117.859 |
| Family | Filobasidiaceae | Var SLA | 0.005 | 0.002 | 41.225 | 2.486 | 0.017 | 65.756 |
| Order | Filobasidiales | Var C | 0.228 | 0.093 | 61.990 | 2.459 | 0.017 | 107.502 |
| Order | Filobasidiales | Var LDMC | 0.000 | 0.000 | 60.385 | 2.421 | 0.018 | 117.859 |
| Order | Filobasidiales | Var SLA | 0.005 | 0.002 | 41.225 | 2.486 | 0.017 | 65.756 |
| Genus | Filobasidium | Var LDMC | 0.000 | 0.000 | 59.167 | 2.391 | 0.020 | 107.850 |
| Genus | Fusicladium | Mean SLA | 0.304 | 0.111 | 19.262 | 2.730 | 0.013 | 114.377 |
| Species | Fusicladium proteae | Mean SLA | 0.304 | 0.111 | 19.262 | 2.730 | 0.013 | 114.377 |
| Genus | Kondoa | Mean CN | -0.013 | 0.004 | 123.431 | -2.828 | 0.005 | -85.870 |
| Genus | Kondoa | Mean LDMC | -0.001 | 0.001 | 103.862 | -2.164 | 0.033 | -41.471 |
| Genus | Kondoa | Mean P | 0.004 | 0.002 | 74.132 | 2.022 | 0.047 | -50.663 |
| Genus | Kondoa | Mean SLA | 0.028 | 0.005 | 130.447 | 5.158 | < 0.001 | -74.733 |
| Genus | Kondoa | Var CN | 0.004 | 0.002 | 163.246 | 2.013 | 0.046 | -80.773 |
| Family | Kondoaceae | Mean CN | -0.059 | 0.022 | 85.321 | -2.686 | 0.009 | 285.913 |
| Family | Kondoaceae | Mean P | 0.019 | 0.009 | 103.228 | 2.145 | 0.034 | 296.641 |
| Family | Kondoaceae | Mean SLA | 0.052 | 0.021 | 84.565 | 2.518 | 0.014 | 223.222 |
| Family | Mycosphaerellaceae | Mean CN | 0.084 | 0.040 | 126.006 | 2.080 | 0.040 | 532.576 |
| Family | Mycosphaerellaceae | Var LDMC | 0.000 | 0.000 | 136.180 | 2.240 | 0.027 | 554.035 |
| Family | Phaeosphaeriaceae | Mean SLA | 0.122 | 0.044 | 35.210 | 2.781 | 0.009 | 144.056 |
| Family | Pleosporaceae | Var C | 0.124 | 0.062 | 151.982 | 1.995 | 0.048 | 359.995 |
| Order | Pleosporales | Var C | 0.368 | 0.175 | 192.924 | 2.100 | 0.037 | 928.411 |
| Genus | Ramularia | Mean CN | 0.072 | 0.023 | 81.792 | 3.116 | 0.003 | 248.385 |
| Genus | Ramularia | Mean N | -0.938 | 0.331 | 59.457 | -2.830 | 0.006 | 259.560 |
| Class | Sordariomycetes | Var SLA | -0.009 | 0.004 | 44.556 | -2.194 | 0.033 | 126.093 |
| Family | Sympoventuriaceae | Mean SLA | 0.304 | 0.111 | 19.262 | 2.730 | 0.013 | 114.377 |
| Species | Taphrina carpini | Mean C | -0.095 | 0.042 | 30.021 | -2.269 | 0.031 | 61.189 |
| Species | Taphrina carpini | Mean NP | -11.312 | 4.852 | 22.050 | -2.331 | 0.029 | 51.024 |
| Species | Taphrina carpini | Mean P | 0.012 | 0.006 | 38.683 | 2.146 | 0.038 | 76.716 |
| Species | Taphrina carpini | Var CN | -0.015 | 0.007 | 45.231 | -2.125 | 0.039 | 78.870 |
| Order | Tremellales | Mean SLA | 0.123 | 0.059 | 105.070 | 2.085 | 0.039 | 799.167 |
| Class | Tremellomycetes | Mean CN | -0.151 | 0.061 | 145.032 | -2.456 | 0.015 | 1026.925 |
| Class | Tremellomycetes | Mean SLA | 0.154 | 0.074 | 127.280 | 2.087 | 0.039 | 938.165 |
| Family | Venturiaceae | Mean P | -0.032 | 0.011 | 15.204 | -2.831 | 0.013 | 44.227 |
| Family | Venturiaceae | Var N | 11.673 | 3.991 | 14.000 | 2.925 | 0.011 | 31.137 |
| Order | Venturiales | Mean SLA | 0.254 | 0.108 | 44.010 | 2.352 | 0.023 | 226.320 |

Table S3: Linear mixed effect model results for trophic modes, guilds and growth forms of fungal endophytes, testing the effect of mean leaf trait values (Mean) and intraindividual trait variation (Var) on ACE, separately for each assigned trophic mode, guild and growth form (Taxon). Shown are estimates and standard errors (se) of marginal effects of fixed factors as well as p-values and Akaike information criterion (AIC). Shown are only effects significant using ACE.

| Type | Taxon | Predictor | Estimate | SE | df | t | P | AIC |
| --- | --- | --- | --- | --- | --- | --- | --- | --- |
| Trophic mode | Pathotroph | Mean LDMC | -0.012 | 0.005 | 156.799 | -2.169 | 0.032 | 841.125 |
| Trophic mode | Pathotroph | Mean SLA | 0.117 | 0.050 | 137.411 | 2.366 | 0.019 | 782.766 |
| Trophic mode | Saprotroph | Mean SLA | 0.155 | 0.063 | 172.228 | 2.455 | 0.015 | 830.970 |
| Trophic mode | Saprotroph-Symbiotroph | Mean LDMC | -0.003 | 0.001 | 48.938 | -2.402 | 0.020 | 246.837 |
| Trophic mode | Saprotroph-Symbiotroph | Mean SLA | 0.033 | 0.013 | 31.108 | 2.541 | 0.016 | 215.164 |
| Guild | Epiphyte-Saprotroph | Mean SLA | 0.016 | 0.008 | 105.798 | 2.063 | 0.042 | 53.747 |
| Guild | Epiphyte-Saprotroph | Var CN | 0.007 | 0.003 | 108.977 | 1.989 | 0.049 | 75.630 |
| Guild | Fungal_Parasite | Var CN | 0.068 | 0.027 | 7.000 | 2.542 | 0.039 | 33.594 |
| Guild | Fungal_Parasite-Saprotroph | Mean SLA | 0.123 | 0.059 | 105.070 | 2.085 | 0.039 | 799.167 |
| Guild | Saprotroph | Mean CN | -0.095 | 0.046 | 178.751 | -2.071 | 0.040 | 821.485 |
| Guild | Saprotroph | Mean SLA | 0.153 | 0.056 | 164.755 | 2.752 | 0.007 | 769.579 |
| Guild | Pathogen | Mean LDMC | -0.010 | 0.005 | 146.566 | -2.011 | 0.046 | 796.083 |
| Guild | Pathogen | Mean SLA | 0.091 | 0.045 | 128.075 | 1.994 | 0.048 | 741.421 |
| Guild | Pathogen-Saprotroph | Var LDMC | 0.000 | 0.000 | 177.182 | 2.053 | 0.041 | 953.833 |
| Growth form | Dimorphic | Mean CN | -0.026 | 0.013 | 82.707 | -2.041 | 0.044 | 128.828 |
| Growth form | Dimorphic | Mean P | 0.010 | 0.005 | 96.195 | 2.042 | 0.044 | 145.915 |
| Growth form | Dimorphic | Mean SLA | 0.045 | 0.013 | 86.865 | 3.550 | 0.001 | 90.762 |
| Growth form | Dimorphic-Facultative Yeast | Mean SLA | 0.098 | 0.042 | 67.079 | 2.351 | 0.022 | 278.315 |
| Growth form | Dimorphic-Facultative Yeast | Var C | 0.643 | 0.168 | 76.001 | 3.840 | < 0.000 | 268.065 |
| Growth form | Tremelloid-Yeast | Mean SLA | 0.123 | 0.059 | 105.070 | 2.085 | 0.039 | 799.167 |
| Growth form | Yeast | Var SLA | 0.008 | 0.003 | 62.894 | 2.546 | 0.013 | 269.057 |

Table S4: PERMANOVA results based on Bray-Curtis-distance, testing the effect of host tree species and mean trait values on fungal endophyte community composition for with different datasets. Datasets are based individual fungal taxa, ranging from the kingdom of fungi, over the two most prominent phyla (Ascomycota, Basidiomycota), to the most prominent classes (Dothideomycetes, Leotiomycetes, Taphrinomycetes and Tremellomycetes) of this study.

| Taxon | Predictor | Degrees of freedom | Sum of squares | R^2^ | F | Pr(>F) |
| --- | --- | --- | --- | --- | --- | --- |
| Fungi | Tree species | 7 | 9.362 | 0.321 | 7.365 | 0.001*** |
| Fungi | Mean SLA | 1 | 0.347 | 0.012 | 1.913 | 0.047* |
| Fungi | Mean LDMC | 1 | 0.363 | 0.012 | 1.999 | 0.033* |
| Fungi | Mean C | 1 | 0.167 | 0.006 | 0.918 | 0.481 |
| Fungi | Mean N | 1 | 0.122 | 0.004 | 0.673 | 0.775 |
| Fungi | Mean P | 1 | 0.180 | 0.006 | 0.990 | 0.404 |
| Fungi | Mean CN | 1 | 0.100 | 0.003 | 0.549 | 0.901 |
| Fungi | Residual | 81 | 14.709 | 0.504 |  |  |
| Fungi | Total | 94 | 29.179 | 1.000 |  |  |
|  |  |  |  |  |  |  |
| Ascomycota | Tree species | 7 | 7.696 | 0.297 | 6.292 | 0.001*** |
| Ascomycota | Mean SLA | 1 | 0.328 | 0.013 | 1.879 | 0.037* |
| Ascomycota | Mean LDMC | 1 | 0.301 | 0.012 | 1.721 | 0.075. |
| Ascomycota | Mean C | 1 | 0.183 | 0.007 | 1.045 | 0.392 |
| Ascomycota | Mean N | 1 | 0.146 | 0.006 | 0.835 | 0.571 |
| Ascomycota | Mean P | 1 | 0.164 | 0.006 | 0.936 | 0.468 |
| Ascomycota | Mean CN | 1 | 0.115 | 0.004 | 0.660 | 0.781 |
| Ascomycota | Residual | 81 | 14.154 | 0.546 |  |  |
| Ascomycota | Total | 94 | 25.914 | 1.000 |  |  |
|  |  |  |  |  |  |  |
| Basidiomycota | Tree species | 7 | 3.290 | 0.134 | 1.783 | 0.001*** |
| Basidiomycota | Mean SLA | 1 | 0.384 | 0.016 | 1.459 | 0.12 |
| Basidiomycota | Mean LDMC | 1 | 0.559 | 0.023 | 2.121 | 0.019* |
| Basidiomycota | Mean C | 1 | 0.307 | 0.012 | 1.166 | 0.264 |
| Basidiomycota | Mean N | 1 | 0.287 | 0.012 | 1.090 | 0.305 |
| Basidiomycota | Mean P | 1 | 0.274 | 0.011 | 1.039 | 0.383 |
| Basidiomycota | Mean CN | 1 | 0.437 | 0.018 | 1.659 | 0.073. |
| Basidiomycota | Residual | 74 | 19.506 | 0.793 |  |  |
| Basidiomycota | Total | 87 | 24.611 | 1.000 |  |  |
|  |  |  |  |  |  |  |
| Dothideomycetes | Tree species | 7 | 6.504 | 0.283 | 5.755 | 0.001*** |
| Dothideomycetes | Mean SLA | 1 | 0.287 | 0.012 | 1.774 | 0.059. |
| Dothideomycetes | Mean LDMC | 1 | 0.246 | 0.011 | 1.521 | 0.127 |
| Dothideomycetes | Mean C | 1 | 0.178 | 0.008 | 1.103 | 0.352 |
| Dothideomycetes | Mean N | 1 | 0.073 | 0.003 | 0.454 | 0.919 |
| Dothideomycetes | Mean P | 1 | 0.204 | 0.009 | 1.264 | 0.218 |
| Dothideomycetes | Mean CN | 1 | 0.064 | 0.003 | 0.394 | 0.957 |
| Dothideomycetes | Residual | 81 | 13.078 | 0.569 |  |  |
| Dothideomycetes | Total | 94 | 22.967 | 1.000 |  |  |
|  |  |  |  |  |  |  |
| Leotiomycetes | Tree species | 7 | 6.739 | 0.249 | 4.350 | 0.001*** |
| Leotiomycetes | Mean SLA | 1 | 0.421 | 0.016 | 1.903 | 0.079. |
| Leotiomycetes | Mean LDMC | 1 | 0.649 | 0.024 | 2.934 | 0.01** |
| Leotiomycetes | Mean C | 1 | 0.248 | 0.009 | 1.122 | 0.32 |
| Leotiomycetes | Mean N | 1 | 0.286 | 0.011 | 1.291 | 0.242 |
| Leotiomycetes | Mean P | 1 | 0.143 | 0.005 | 0.644 | 0.72 |
| Leotiomycetes | Mean CN | 1 | 0.234 | 0.009 | 1.055 | 0.359 |
| Leotiomycetes | Residual | 73 | 16.157 | 0.598 |  |  |
| Leotiomycetes | Total | 86 | 27.037 | 1.000 |  |  |
|  |  |  |  |  |  |  |
| Tremellomycetes | Tree species | 7 | 3.063 | 0.145 | 1.883 | 0.001*** |
| Tremellomycetes | Mean SLA | 1 | 0.293 | 0.014 | 1.260 | 0.243 |
| Tremellomycetes | Mean LDMC | 1 | 0.308 | 0.015 | 1.324 | 0.215 |
| Tremellomycetes | Mean C | 1 | 0.301 | 0.014 | 1.295 | 0.243 |
| Tremellomycetes | Mean N | 1 | 0.341 | 0.016 | 1.468 | 0.153 |
| Tremellomycetes | Mean P | 1 | 0.148 | 0.007 | 0.635 | 0.754 |
| Tremellomycetes | Mean CN | 1 | 0.524 | 0.025 | 2.255 | 0.025* |
| Tremellomycetes | Residual | 70 | 16.267 | 0.769 |  |  |
| Tremellomycetes | Total | 83 | 21.142 | 1.000 |  |  |
|  |  |  |  |  |  |  |
| Taphrinomycetes | Tree species | 7 | 1.743 | 0.121 | 1.490 | 0.061. |
| Taphrinomycetes | Mean SLA | 1 | 0.342 | 0.024 | 2.045 | 0.099. |
| Taphrinomycetes | Mean LDMC | 1 | 0.204 | 0.014 | 1.218 | 0.287 |
| Taphrinomycetes | Mean C | 1 | 0.131 | 0.009 | 0.781 | 0.543 |
| Taphrinomycetes | Mean N | 1 | 0.149 | 0.010 | 0.889 | 0.458 |
| Taphrinomycetes | Mean P | 1 | 0.152 | 0.010 | 0.906 | 0.455 |
| Taphrinomycetes | Mean CN | 1 | 0.064 | 0.004 | 0.383 | 0.821 |
| Taphrinomycetes | Residual | 69 | 11.533 | 0.798 |  |  |
| Taphrinomycetes | Total | 82 | 14.445 | 1.000 |  |  |

Table S5: PERMANOVA results based on Bray-Curtis-distance, testing the effect of host tree species and intraindividual trait variation on fungal endophyte community composition for with different datasets. Datasets are based individual fungal taxa, ranging from the kingdom of fungi, over the two most prominent phyla (Ascomycota, Basidiomycota), to the most prominent classes (Dothideomycetes, Leotiomycetes, Taphrinomycetes and Tremellomycetes) of this study.

| Taxon | Predictor | Degrees of freedom | Sum of squares | R^2^ | F | Pr(>F) |
| --- | --- | --- | --- | --- | --- | --- |
| Fungi | Tree species | 7 | 11.419 | 0.391 | 8.764 | 0.001*** |
| Fungi | Var LDMC | 1 | 0.314 | 0.011 | 1.689 | 0.075. |
| Fungi | Var C | 1 | 0.087 | 0.003 | 0.469 | 0.936 |
| Fungi | Var SLA | 1 | 0.189 | 0.006 | 1.013 | 0.404 |
| Fungi | Var N | 1 | 0.211 | 0.007 | 1.136 | 0.298 |
| Fungi | Var P | 1 | 0.215 | 0.007 | 1.156 | 0.278 |
| Fungi | Residual | 82 | 15.264 | 0.523 |  |  |
| Fungi | Total | 94 | 29.179 | 1.000 |  |  |
|  |  |  |  |  |  |  |
| Ascomycota | Tree species | 7 | 9.421 | 0.364 | 7.567 | 0.001*** |
| Ascomycota | Var LDMC | 1 | 0.267 | 0.010 | 1.500 | 0.118 |
| Ascomycota | Var C | 1 | 0.133 | 0.005 | 0.749 | 0.676 |
| Ascomycota | Var SLA | 1 | 0.189 | 0.007 | 1.061 | 0.368 |
| Ascomycota | Var N | 1 | 0.186 | 0.007 | 1.047 | 0.374 |
| Ascomycota | Var P | 1 | 0.188 | 0.007 | 1.057 | 0.397 |
| Ascomycota | Residual | 82 | 14.585 | 0.563 |  |  |
| Ascomycota | Total | 94 | 25.914 | 1.000 |  |  |
|  |  |  |  |  |  |  |
| Basidiomycota | Tree species | 7 | 2.615 | 0.106 | 1.347 | 0.033* |
| Basidiomycota | Var LDMC | 1 | 0.160 | 0.007 | 0.578 | 0.884 |
| Basidiomycota | Var C | 1 | 0.134 | 0.005 | 0.482 | 0.912 |
| Basidiomycota | Var SLA | 1 | 0.166 | 0.007 | 0.600 | 0.865 |
| Basidiomycota | Var N | 1 | 0.294 | 0.012 | 1.060 | 0.365 |
| Basidiomycota | Var P | 1 | 0.281 | 0.011 | 1.015 | 0.387 |
| Basidiomycota | Residual | 75 | 20.800 | 0.845 |  |  |
|  | Total | 87 | 24.611 | 1.000 |  |  |
|  |  |  |  |  |  |  |
| Dothideomycetes | Tree species | 7 | 7.979 | 0.347 | 7.003 | 0.001*** |
| Dothideomycetes | Var LDMC | 1 | 0.300 | 0.013 | 1.845 | 0.05* |
| Dothideomycetes | Var C | 1 | 0.149 | 0.006 | 0.914 | 0.484 |
| Dothideomycetes | Var SLA | 1 | 0.176 | 0.008 | 1.081 | 0.366 |
| Dothideomycetes | Var N | 1 | 0.143 | 0.006 | 0.876 | 0.508 |
| Dothideomycetes | Var P | 1 | 0.252 | 0.011 | 1.550 | 0.118 |
| Dothideomycetes | Residual | 82 | 13.346 | 0.581 |  |  |
| Dothideomycetes | Total | 94 | 22.967 | 1.000 |  |  |
|  |  |  |  |  |  |  |
| Leotiomycetes | Tree species | 7 | 8.172 | 0.302 | 5.052 | 0.001*** |
| Leotiomycetes | Var LDMC | 1 | 0.327 | 0.012 | 1.416 | 0.176 |
| Leotiomycetes | Var C | 1 | 0.041 | 0.002 | 0.176 | 0.981 |
| Leotiomycetes | Var SLA | 1 | 0.268 | 0.010 | 1.161 | 0.321 |
| Leotiomycetes | Var N | 1 | 0.215 | 0.008 | 0.928 | 0.458 |
| Leotiomycetes | Var P | 1 | 0.195 | 0.007 | 0.843 | 0.552 |
| Leotiomycetes | Residual | 74 | 17.102 | 0.633 |  |  |
| Leotiomycetes | Total | 86 | 27.037 | 1.000 |  |  |
|  |  |  |  |  |  |  |
| Tremellomycetes | Tree species | 7 | 2.476 | 0.117 | 1.425 | 0.039* |
| Tremellomycetes | Var LDMC | 1 | 0.059 | 0.003 | 0.236 | 0.985 |
| Tremellomycetes | Var C | 1 | 0.125 | 0.006 | 0.502 | 0.836 |
| Tremellomycetes | Var SLA | 1 | 0.139 | 0.007 | 0.560 | 0.798 |
| Tremellomycetes | Var N | 1 | 0.155 | 0.007 | 0.624 | 0.734 |
| Tremellomycetes | Var P | 1 | 0.237 | 0.011 | 0.954 | 0.478 |
| Tremellomycetes | Residual | 71 | 17.627 | 0.834 |  |  |
| Tremellomycetes | Total | 83 | 21.142 | 1.000 |  |  |
|  |  |  |  |  |  |  |
| Taphrinomycetes | Tree species | 7 | 1.529 | 0.106 | 1.257 | 0.172 |
| Taphrinomycetes | Var LDMC | 1 | 0.137 | 0.010 | 0.790 | 0.548 |
| Taphrinomycetes | Var C | 1 | 0.187 | 0.013 | 1.073 | 0.359 |
| Taphrinomycetes | Var SLA | 1 | 0.075 | 0.005 | 0.429 | 0.805 |
| Taphrinomycetes | Var N | 1 | 0.047 | 0.003 | 0.270 | 0.907 |
| Taphrinomycetes | Var P | 1 | 0.037 | 0.003 | 0.212 | 0.936 |
| Taphrinomycetes | Residual | 70 | 12.168 | 0.842 |  |  |
| Taphrinomycetes | Total | 82 | 14.445 | 1.000 |  |  |

Table S6: Results for Moran’s *I* and Blomberg’s *K* using model slopes from the linear mixed effects models (Table S2 ) of individual mean leaf traits values for throughout taxonomic ranks ranging from class to species testing for a phylogenetic signal. Statistically significant p-values are highlighted bold.

| Rank | Trait | *I* | *I* expected | standard deviation *I* | p-value *I* | *K* | p-value *K* |
| --- | --- | --- | --- | --- | --- | --- | --- |
| Class | SLA | -0.115 | -0.143 | 0.032 | 0.382 | 0.918 | 0.284 |
| Class | LDMC | -0.181 | -0.143 | 0.033 | 0.250 | 0.515 | 0.963 |
| Class | CN | -0.117 | -0.143 | 0.028 | 0.356 | 0.997 | 0.214 |
| Class | C | -0.192 | -0.143 | 0.026 | 0.058 | 0.474 | 0.970 |
| Class | N | -0.120 | -0.143 | 0.034 | 0.517 | 1.007 | 0.187 |
| Class | P | -0.188 | -0.143 | 0.028 | 0.116 | 0.481 | 0.981 |
| Class | CP | -0.181 | -0.143 | 0.025 | 0.131 | 0.476 | 0.966 |
| Class | NP | -0.176 | -0.143 | 0.021 | 0.109 | 0.500 | 0.973 |
| Order | SLA | -0.108 | -0.091 | 0.023 | 0.455 | 0.561 | 0.759 |
| Order | LDMC | -0.082 | -0.091 | 0.024 | 0.699 | 0.960 | 0.115 |
| Order | CN | -0.068 | -0.091 | 0.024 | 0.348 | 0.914 | 0.128 |
| Order | C | -0.105 | -0.091 | 0.024 | 0.562 | 0.627 | 0.597 |
| Order | N | -0.089 | -0.091 | 0.024 | 0.934 | 0.730 | 0.306 |
| Order | P | -0.114 | -0.091 | 0.022 | 0.297 | 0.510 | 0.861 |
| Order | CP | -0.107 | -0.091 | 0.024 | 0.497 | 0.510 | 0.894 |
| Order | NP | -0.118 | -0.091 | 0.022 | 0.233 | 0.483 | 0.903 |
| Family | SLA | -0.171 | -0.083 | 0.082 | 0.286 | 0.094 | 0.825 |
| Family | LDMC | -0.205 | -0.083 | 0.060 | **0.044** | 0.081 | 0.827 |
| Family | CN | -0.131 | -0.083 | 0.104 | 0.646 | 0.085 | 0.940 |
| Family | C | -0.185 | -0.083 | 0.065 | 0.119 | 0.085 | 0.867 |
| Family | N | -0.209 | -0.083 | 0.093 | 0.177 | 0.081 | 0.903 |
| Family | P | -0.473 | -0.083 | 0.090 | **< 0.001** | 0.055 | 0.973 |
| Family | CP | -0.329 | -0.083 | 0.062 | **< 0.001** | 0.064 | 0.966 |
| Family | NP | 0.053 | -0.083 | 0.108 | 0.205 | 0.240 | 0.588 |
| Genus | SLA | -0.230 | -0.100 | 0.055 | **0.019** | 0.094 | 0.130 |
| Genus | LDMC | -0.209 | -0.100 | 0.067 | 0.103 | 0.083 | 0.166 |
| Genus | CN | -0.217 | -0.100 | 0.099 | 0.237 | 0.186 | **0.001** |
| Genus | C | -0.348 | -0.100 | 0.096 | **0.010** | 0.108 | **0.001** |
| Genus | N | -0.268 | -0.100 | 0.095 | 0.077 | 0.156 | **0.001** |
| Genus | P | -0.394 | -0.100 | 0.082 | **< 0.001** | 0.074 | 0.078 |
| Genus | CP | -0.251 | -0.100 | 0.053 | **0.004** | 0.081 | 0.184 |
| Genus | NP | -0.146 | -0.100 | 0.096 | 0.633 | 0.128 | **0.001** |
| Species | SLA | -0.173 | -0.083 | 0.050 | 0.072 | 0.078 | 0.878 |
| Species | LDMC | -0.181 | -0.083 | 0.063 | 0.123 | 0.081 | 0.909 |
| Species | CN | -0.196 | -0.083 | 0.097 | 0.246 | 0.092 | 0.923 |
| Species | C | -0.326 | -0.083 | 0.098 | **0.013** | 0.067 | 0.980 |
| Species | N | -0.226 | -0.083 | 0.093 | 0.124 | 0.086 | 0.939 |
| Species | P | -0.337 | -0.083 | 0.080 | **0.002** | 0.064 | 0.985 |
| Species | CP | -0.223 | -0.083 | 0.051 | **0.007** | 0.073 | 0.938 |
| Species | NP | -0.048 | -0.083 | 0.101 | 0.729 | 0.110 | 0.819 |

Table S7: Results of Moran’s *I* and Blomberg’s *K* analyses testing for phylogenetic signal in host leaf trait values. Trait means were calculated per ASV species and aggregated across taxonomic ranks from ASV to Class. Statistically significant p-values are shown in bold**.**

| Rank | Trait | *I* | *I* expected | standard deviation *I* | p-value *I* | *K* | p-value *K* |
| --- | --- | --- | --- | --- | --- | --- | --- |
| ASV | SLA | 0.018 | -0.001 | 0.008 | **0.011** | 0.000 | 0.367 |
| ASV | LDMC | 0.056 | -0.001 | 0.008 | **< 0.001** | 0.000 | 0.068 |
| ASV | CN | 0.008 | -0.001 | 0.008 | 0.247 | 0.000 | 0.385 |
| ASV | C | 0.059 | -0.001 | 0.008 | **< 0.001** | 0.000 | 0.302 |
| ASV | N | 0.010 | -0.001 | 0.008 | 0.157 | 0.000 | 0.350 |
| ASV | P | 0.031 | -0.001 | 0.008 | **< 0.001** | 0.000 | 0.381 |
| ASV | CP | 0.035 | -0.001 | 0.008 | **< 0.001** | 0.000 | **0.028** |
| ASV | NP | 0.050 | -0.001 | 0.008 | **< 0.001** | 0.000 | 0.112 |
| Genus | SLA | -0.030 | -0.004 | 0.016 | 0.099 | 0.043 | 0.347 |
| Genus | LDMC | -0.007 | -0.004 | 0.016 | 0.854 | 0.052 | 0.114 |
| Genus | CN | -0.018 | -0.004 | 0.016 | 0.397 | 0.059 | **0.047** |
| Genus | C | -0.014 | -0.004 | 0.016 | 0.529 | 0.030 | 0.719 |
| Genus | N | 0.017 | -0.004 | 0.016 | 0.180 | 0.104 | **0.001** |
| Genus | P | 0.012 | -0.004 | 0.016 | 0.290 | 0.047 | 0.263 |
| Genus | CP | 0.017 | -0.004 | 0.015 | 0.161 | 0.065 | 0.052 |
| Genus | NP | -0.005 | -0.004 | 0.015 | 0.953 | 0.047 | 0.378 |
| Species | SLA | -0.030 | -0.004 | 0.016 | 0.099 | 0.043 | 0.373 |
| Species | LDMC | -0.007 | -0.004 | 0.016 | 0.854 | 0.052 | 0.128 |
| Species | CN | -0.018 | -0.004 | 0.016 | 0.397 | 0.059 | **0.049** |
| Species | C | -0.014 | -0.004 | 0.016 | 0.529 | 0.030 | 0.753 |
| Species | N | 0.017 | -0.004 | 0.016 | 0.180 | 0.104 | **0.001** |
| Species | P | 0.012 | -0.004 | 0.016 | 0.290 | 0.047 | 0.248 |
| Species | CP | 0.017 | -0.004 | 0.015 | 0.161 | 0.065 | 0.051 |
| Species | NP | -0.005 | -0.004 | 0.015 | 0.953 | 0.047 | 0.365 |
| Family | SLA | -0.013 | -0.010 | 0.012 | 0.835 | 0.266 | 0.387 |
| Family | LDMC | 0.003 | -0.010 | 0.012 | 0.258 | 0.488 | **0.001** |
| Family | CN | -0.009 | -0.010 | 0.012 | 0.893 | 0.432 | **0.007** |
| Family | C | -0.012 | -0.010 | 0.012 | 0.893 | 0.370 | **0.047** |
| Family | N | -0.008 | -0.010 | 0.012 | 0.817 | 0.437 | **0.009** |
| Family | P | -0.001 | -0.010 | 0.012 | 0.431 | 0.310 | 0.139 |
| Family | CP | 0.001 | -0.010 | 0.012 | 0.344 | 0.427 | **0.018** |
| Family | NP | -0.008 | -0.010 | 0.010 | 0.828 | 0.157 | 0.842 |
| Order | SLA | -0.025 | -0.018 | 0.010 | 0.480 | 0.380 | 0.699 |
| Order | LDMC | -0.021 | -0.018 | 0.010 | 0.716 | 0.481 | 0.252 |
| Order | CN | 0.014 | -0.018 | 0.010 | **0.002** | 0.433 | 0.400 |
| Order | C | -0.028 | -0.018 | 0.010 | 0.306 | 0.347 | 0.786 |
| Order | N | -0.005 | -0.018 | 0.010 | 0.223 | 0.572 | 0.070 |
| Order | P | -0.020 | -0.018 | 0.010 | 0.784 | 0.485 | 0.245 |
| Order | CP | -0.019 | -0.018 | 0.010 | 0.878 | 0.478 | 0.298 |
| Order | NP | -0.018 | -0.018 | 0.010 | 0.932 | 0.558 | 0.069 |
| Class | SLA | -0.047 | -0.048 | 0.014 | 0.987 | 0.637 | 0.552 |
| Class | LDMC | -0.054 | -0.048 | 0.011 | 0.586 | 0.628 | 0.587 |
| Class | CN | -0.028 | -0.048 | 0.014 | 0.156 | 0.764 | 0.275 |
| Class | C | -0.041 | -0.048 | 0.014 | 0.654 | 0.706 | 0.377 |
| Class | N | -0.048 | -0.048 | 0.013 | 0.957 | 0.674 | 0.448 |
| Class | P | -0.044 | -0.048 | 0.014 | 0.796 | 0.742 | 0.284 |
| Class | CP | -0.048 | -0.048 | 0.012 | 0.974 | 0.617 | 0.614 |
| Class | NP | -0.044 | -0.048 | 0.013 | 0.801 | 0.856 | 0.173 |
